# Biomedical Applications of Metal–Organic Frameworks Revisited

**DOI:** 10.1021/acs.iecr.4c03698

**Published:** 2025-01-14

**Authors:** Pelin Sezgin, Ezgi Gulcay-Ozcan, Marija Vučkovski, Aleksandra M. Bondžić, Ilknur Erucar, Seda Keskin

**Affiliations:** † Koç University, Department of Chemical and Biological Engineering, 34450 Istanbul, Turkey; ‡ Sabanci University, Faculty of Engineering and Natural Sciences, Istanbul 34956, Turkey; § Vinča Institute of Nuclear Sciences, National Institute of the Republic of Serbia, University of Belgrade, P.O. Box 522, 11000 Belgrade, Serbia; ∥ Ozyegin University, Department of Natural and Mathematical Sciences, Faculty of Engineering, 34794 Istanbul, Turkey

## Abstract

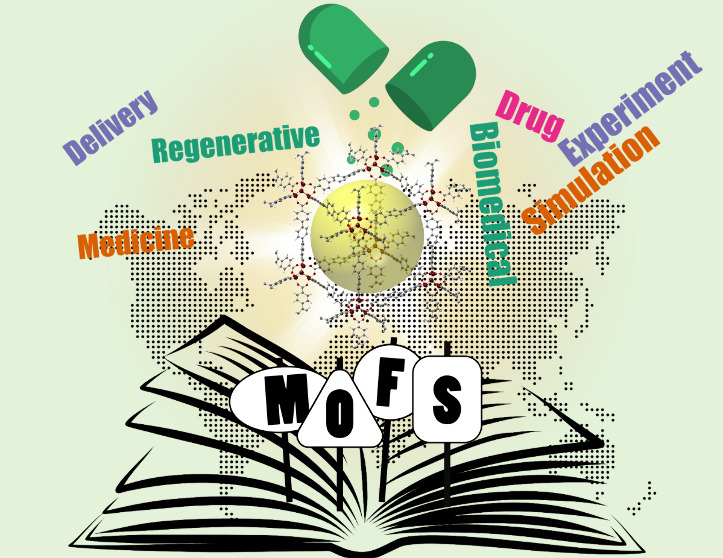

Metal–organic frameworks (MOFs) have been shown to be great alternatives to traditional porous materials in various chemical applications, and they have been very widely studied for biomedical applications in the past decade specifically for drug storage. After our review published in 2011 [Keskin and Kızılel, *Ind*. *Eng*. *Chem*. *Res*. 2011, 50 (4), 1799–1812, 10.1021/ie101312k], we have witnessed a very fast growth not only in the number and variety of MOFs but also in their usage across a broad spectrum of biomedical fields. With the recent integration of molecular modeling and data science approaches to the experimental studies, biomedical applications of MOFs have been significantly accelerated positioning them as pivotal components in the regenerative medicine, medical imaging, and diagnostics. In this review, we visited the diverse biomedical applications of MOFs considering the recent experimental and computational efforts on drug storage and delivery, bioimaging, and biosensing. We focused on the underlying mechanisms governing the molecular interactions between MOFs and biological systems and discussed both the opportunities and challenges in the field to highlight the potential of MOFs in advanced therapeutics for cancer and neurological diseases.

## 1 Background

**Figure 1:**
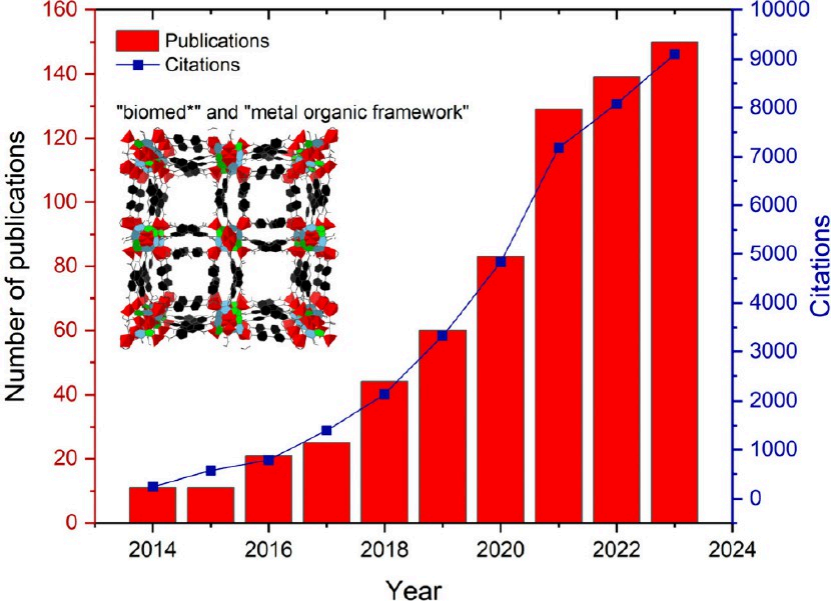
Number of publications and their citations featuring the term “biomed*” and “metal organic framework” in their topics. Accessed: 2024–09–17 at Web of Science.

Metal–organic frameworks (MOFs), consisting of metal ions or clusters bridged by organic ligands, have emerged as a novel class of porous materials with diverse applications ranging from gas storage and separation^1^ to catalysis^2^ and sensing.^3^ Structural properties of MOFs, such as ultrahigh porosities, large surface areas, tunable pore sizes and shapes, and the large variety in their chemical functionalities, have attracted researchers’ interest in the biomedical field to address the current challenges in drug encapsulation and delivery, imaging, and biosensing.^4^ As a proof of this, the significant increase in the number of publications on biomedical applications of MOFs can be seen in Figure 1, with around 740 publications between 2014 and 2024 and a total of 41,601 citations by 2024, indicating the rapidly expanding field of research.

**Figure 2:**
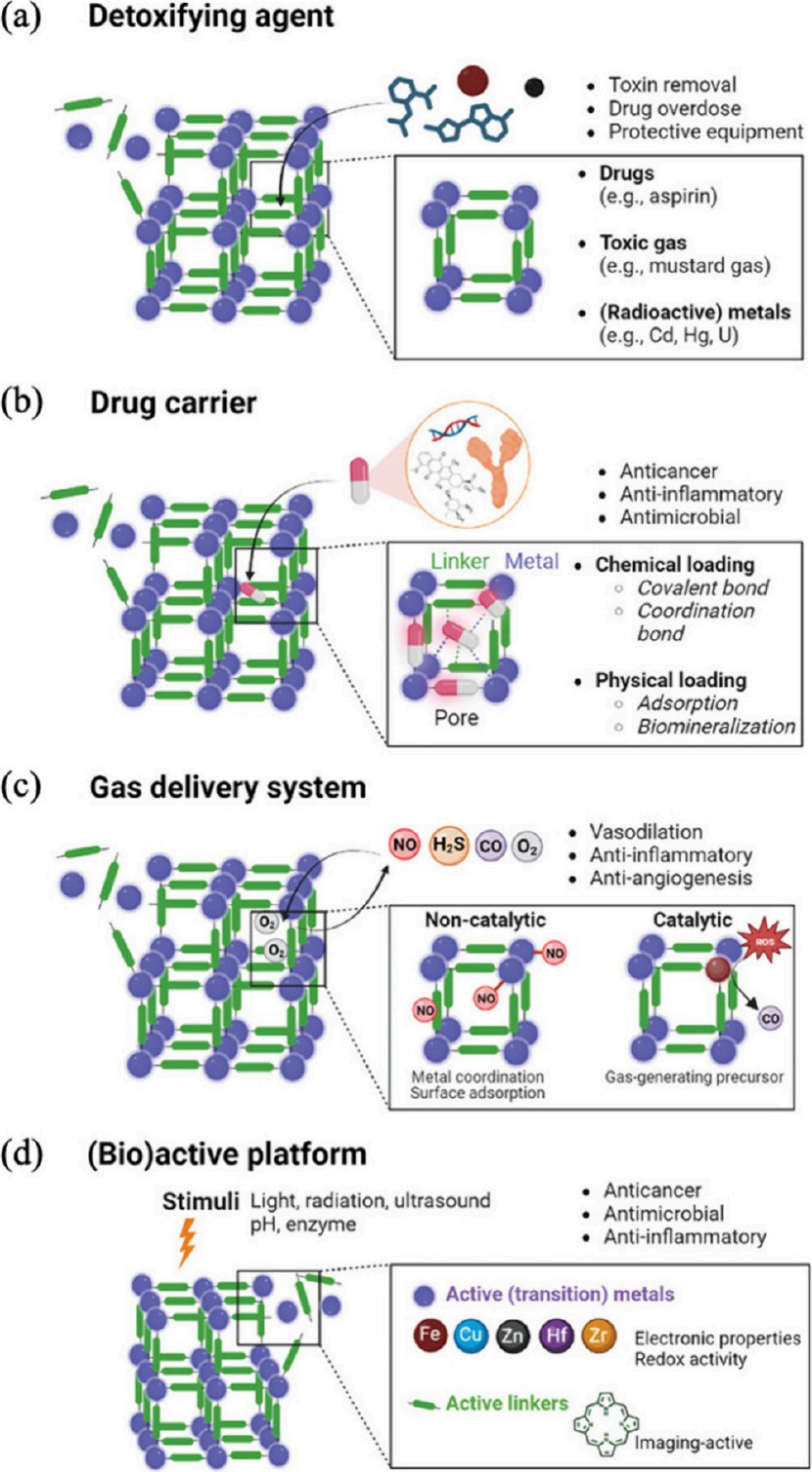
Usage of MOFs in biomedical applications as (a) detoxifying/capturing agents, (b) drug carrier systems, (c) gas delivery systems, and (d) bioactive platforms. Adapted with permission from work by Wang et al.^4^ Available under the Creative Commons Attribution-NonCommercial License. Copyright 2024, Wiley.

By using the reticular design principles, MOFs can be modified for specific biomedical applications, especially for encapsulating and delivering therapeutic agents. One of the most compelling aspects of MOFs in the biomedical field is their versatility in functionalization, allowing for the integration of targeted ligands, stimulatory components, and imaging probes, among other functionalities, into the scaffold structure. This capability not only facilitates the selective recognition and binding of biomolecules but also enables the monitoring of biological processes in real time, paving the way for personalized and precise medical approaches. Figure 2 shows the main use of MOFs in biomedicine as (a) detoxifying/capturing agents, (b) drug carriers, (c) gas delivery systems, and (d) bioactive platforms.

The biodegradability and biocompatibility of MOFs, combined with their ability to modulate the drug release kinetics and pharmacokinetics, hold enormous potential for improving the safety and efficacy of therapeutic interventions. MOF-based drug carrier systems offer innovative solutions to challenges such as drug resistance, poor bioavailability, and systemic toxicity, revolutionizing the landscape of drug delivery and therapy.^5^ Since MOFs have tunable physical and chemical pore environment, biomedical gas molecules,^6,7^ such as NO, CO, H_2_S, and O_2_, uremic toxins, and radioactive metals can be entrapped into their pores via physisorption or chemisorption. Highly porous structures of MOFs make them ideal candidates as drug carriers. MOFs not only increase solubility and bioavailability of the drugs but also improve their efficacy. In addition, MOFs enable precise control of drug release, leading to localized delivery. MOF-based drug delivery systems also show significant promise when exposed to environmental stimuli, such as pH, light, temperature, or the presence of certain compounds. For example, a pH-responsive MOF can release its cargo in acidic environments such as tumor tissues, where the pH is lower compared to normal tissues.^8^ This targeted and on-demand drug release mechanism can enhance therapeutic outcomes and minimize systemic exposure to the drug. Stimuli-responsive MOF-based drug delivery systems represent a promising platform for personalized treatment strategies, as detailed in the following section.

Back in 2011, we reviewed the biomedical applications of MOFs^9^ by focusing on their high drug loading capacity and discussed the challenges related to their stability, toxicology, and biocompatibility to make them as viable therapeutics options for the future. Since then, we have witnessed quick growth not only in the number and type of synthesized MOFs but also in the number of studies revealing the potential of MOFs in various biomedical applications. Among more than 125,383 synthesized MOFs which were collected from the Cambridge Structural Database (CSD) using the Conquest^10^ tool on September 17, 2024, the current challenge is to select the most appropriate MOF for a target drug delivery. MOFs need to be nontoxic and possess the appropriate pore size for the loading and delivery of the drugs in addition to being stable and durable under physiological conditions. Additional requirements can include specific functionalization to create certain binding sites^11^ or enable signal transduction for sensing and imaging.^12^

Identification of the best MOFs that can satisfy these requirements by performing iterative experiments is not practical, whereas computational modeling has played a significant role in accelerating the use of MOFs for biomedical applications. Molecular modeling of MOFs and biological components, such as drug molecules, enzymes, and proteins, allows us to predict the behavior and performance of MOFs in complex biological environments. Very recently, artificial intelligence (AI) and more specifically machine learning (ML) methods have allowed researcher to analyze large data sets of MOFs to develop useful structure–performance relationships to optimize the structural and chemical properties of MOFs for a wide range of biomedical applications. The integration of AI-assisted computational modeling to experimental research has a great potential to design high-performing MOFs for more targeted and effective treatments in healthcare. As a result of these rapid developments, we decided to revisit the topic of biomedical applications of MOFs to discuss how the field has evolved within the last 13 years and to highlight the new directions. We focused on recent experimental studies on MOFs for drug storage/delivery and sensing and computational studies that underpin utility of MOFs for these applications. Through a comprehensive examination of recent opportunities and challenges, we aim to shed light on the transformative impact of MOFs on biomedical research and their prospects for translation into clinical practice.

## 2 MOFs as Drug Loading and Delivery Systems

Despite the favorable properties of MOFs mentioned above, they often need to be functionalized and modified to improve drug loading, selective targeting, and stability in the biological environment due to their low physiological stability, tendency to aggregate, and lack of selectivity with respect to target sites.^4^ These modifications can be performed during their synthesis or by postsynthesis, allowing MOFs to load and release therapeutic agents in a controlled manner. Modifying the surface of MOFs with biocompatible molecules (polymers, liposomes, and biomolecules) prevents their aggregation and improves their stability in physiological medium, increasing their drug delivery capability. On the other hand, selective targeting can be achieved by designing MOFs which can respond to different stimuli.^13,14^ This enables controlled drug release triggered by internal factors such as pH, shear stress, or enzymes as well as external factors such as ultrasound and magnetism. Controlled release of therapeutics from the pores of the MOFs can also be achieved by modifying the MOFs with suitable ligand components. Recent studies on modified MOFs as drug carriers for the treatment of cancer and neurological diseases are summarized in Table 1. Most of these studies have focused on the drug delivery for cancer, but it is worth noting that MOFs are also promising agents for use in Alzheimer’s disease (AD), antimicrobial therapy, and viral diseases.^15,16^

**Table 1:** Recent Studies on MOF-Based Drug Carriers for the Treatment of Cancer and Neurological Diseases^a^

	MOF delivery platform	Components	Drug	Stimuli type	Ref
Cancer	Fe_3_O_4_@C_12_H_22_O_11_@MIL-88-DOX	Fe_3_O_4_, MIL-88, maltose, folic acid conjugated chitosan	DOX	pH	(17)
	Drug@PCN-224-FA	PCN-224, folic acid	DOX or CPT	pH, laser irradiation	(18)
	AuNCs@ZIF-8-DOX	ZIF-8, AuNCs	DOX	pH, laser irradiation	(19)
	SiO_2_@Fe_3_O_4_–HA-MIL-100-GQDs	MIL-100, hydroxyapatite, Fe_3_O_4_, graphene quantum dots	DOX	pH	(20)
	F127@DOX@MIL-100	MIL-100; poly(ethylene glycol-*co*-propylene glycol)	DOX	GSH and ATP	(21)
	ZIF-8@Fe_3_O_4_	carboxymethyl cellulose, ZIF-8, Fe_3_O_4_	DOX	pH	(22)
	ZIF-8@DOX@porphyrinic Zr MOF	ZIF-8, porphyrinic Zr MOF	DOX	pH	(23)
	AR-ZS/ID-P NPs	ZIF-8; AR peptide; SF	DOX, ICG	pH, NIR laser irradiation	(24)
	TD@Z	ZIF-8, DOX, thrombin	DOX, thrombin	pH	(25)
	DOX@Fe@ZIF-8	ZIF-8; Fe; DOX	DOX	pH	(26)
	CaO_2_-DOX-CuMOF/PEG	CaO_2_; CuMOF; PEG; DOX	DOX	GSH	(27)
	DOX@Ag-MOF	DOX, AgMOF	DOX	pH	(28)
	5-FU@bi-MIL88B-FC	(FeCo) MIL-88; folic acid, 5-FU	5-FU	pH	(29)
	MIL-101(Fe)@5-FU@FA	Fe MIL-101; folic acid, 5-FU	5-FU	pH	(30)
	5-FU@ZIF-8-SDG	ZIF-8; hyaluronic acid-hydroxypropyl methyl cellulose	5-FU; SDG	pH	(31)
Neurological disorder	CD-MOF	cyclodextrin-based potassium MOF	huperzine A	Dissolving in nasal mucosa	(32)
	ZIF-8@alginate	ZIF-8, alginate	metformin	pH	(33)
	(Fe)MIL-100-Met@alginate	Metformin (Met); MIL-100, alginate	metformin	pH	(34)
	PMP@MIL-88A	PMP; MIL-88A	Dopamine	Magneto-phoretic guidance	(35)

a MIL: Matériaux de l’Institut Lavoisier, DOX: doxorubicin, PCN: porous coordination network, CPT: camptothecin, ZIF: zeolitic imidazolate framework, SF: silk fibroin, ICG: indocyanine green, FU: fluorouracil, SDG: sonidegib as a therapeutic agent, PMP: carboxyl-functionalized polymeric magnetic particles.

Table 1 summarizes recently published studies on the application of MOFs as drug delivery platforms for cancer and neurological diseases. It is evident that MIL(Fe) (MIL: Matériaux de l’Institut Lavoisier) and ZIF (Zn) (ZIF: Zeolitic Imidazolate Frameworks, a group of MOFs that are topologically isomorphic with zeolites) materials have great potential for encapsulating model anticancer drugs, such as doxorubicin (DOX) and 5-fluorouracil (5-FU), thanks to their biocompatibility and low toxicity. Additionally, cyclodextrin MOFs (CD-MOFs) also stand out. Depending on the type of MOF, different stabilization and stimuli-responsive components were employed to modify the MOFs, with the aim of enhancing the stability of the delivery platform and achieving controlled drug release. For example, Sivakumar et al.^36^ demonstrated the importance of coating Fe-MIL-88B with chitosan for the controlled release of DOX. The authors developed a nanoscale DOX delivery platform by modifying Fe-MIL-88B with NH_2_ groups to stabilize the MOF and improve the loading efficiency of DOX. Chitosan was used as a coating agent to prevent the aggregation of nanoscale Fe-MIL-88B-NH_2_ and to enable pH-responsive DOX release. Based on fluorescence measurements, the authors calculated a 97% DOX loading efficiency with a 6:1 MOF ratio. Prior to the chitosan coating, DOX release reached 97% at pH 5.0 and 83% at pH 7.5 within 2 h. However, after chitosan coating, the amount of DOX released at pH 7.5 significantly decreased to 35%, while the release at pH 5.0 remained unchanged. The authors attributed these results to differences in the protonation state of chitosan, which depend on the pH of the medium.

In a recently published work, Namazi et al. designed a drug delivery platform based on magnetic Fe_3_O_4_ and maltose (C)-modified MOF, MIL-88, for the targeted and pH-controlled release of DOX.^17^ The obtained Fe_3_O_4_@C@MIL-88 was decorated with folic acid-conjugated chitosan (FC), and this coating enabled improved biocompatibility, high cellular permeability, and targeted and controlled release of DOX. Aggregation of Fe_3_O_4_ was avoided, and drug stability was improved by modification with MIL-88 and maltose. Both the drug encapsulation efficiency (EE%) and drug loading capacity (DLC%) of DOX in the Fe_3_O_4_@C@MIL-88 were reported to be high, at 83.6 and 8.3 wt %, respectively. On the other hand, the combination of MIL-88’s pH-response and the FC layer’s sensitivity to acidic pH media resulted in a higher release of DOX at pH 5 than previous MOF carriers used for DOX administration.^18,19^

Among iron-based MOFs, MIL-100 (Fe) is the most extensively studied as a drug carrier thanks to its high uptake capacity and low cytotoxicity. Bhattacharjee et al. have several works on the incorporation of metal nanoparticles into MIL-100 (Fe) to enhance its DOX loading capacity and optimized the DOX release within the tumor microenvironment.^37−39^ They demonstrated that incorporating Fe_3_O_4_ nanoparticles into the pores of MIL-100(Fe) can double the DOX loading capacity compared to bare Fe_3_O_4_ or MIL-100 alone. However, the loading capacity depends on the amount of Fe_3_O_4_ integrated into the MOF.^37^ Authors then integrated ZnO nanoparticles into MIL-100(Fe) and evaluated its DOX loading and release capacity.^38^ They found that the DOX loading capacity depends on the MOF synthesis route, with HF-free methods producing larger mesopores and HF methods smaller ones. ZnO incorporation improved the DOX loading in low-mesopore MOFs but slowed release. They also studied the impact of graphene oxide (GO) incorporation on DOX loading in MIL-100(Fe) and found the highest DOX loading efficiency (29.91%) with 0.5 wt % GO, nearly doubling that of pristine MIL-100(Fe) (16.64%).^39^ Hydrothermal synthesis further improved loading compared to that of room-temperature methods. GO@MIL-100 reduced the initial burst release (10% in 5 h vs 20% for GO) and ensured sustained release (70.96% over 25 days), balancing overdose risk with long-term treatment. Cytotoxicity studies confirmed its safety for HEK293 cells and effectiveness against A549 carcinoma cells.

Karimi et al.^20^ investigated a MOF-based DOX delivery platform obtained by combining MIL-100, hydroxyapatite (HA), and magnetic Fe_3_O_4_ nanoparticles and additionally surface-functionalized with graphene quantum dots (GQDs), SiO_2_@Fe_3_O_4_-HA-MIL-100-GQDs. This modification provided drastically enhanced stability, biocompatibility, and fluorescence in addition to strong antioxidant and anticancer properties. The antitumor activity was achieved by the controlled release of DOX under acidic conditions, indicating a pH-responsive drug delivery platform.

In addition to acidic pH, high concentrations of glutathione (GSH) and adenosine-5′-triphosphate (ATP) are also commonly used stimuli for the targeted release of anticancer drugs from many drug delivery platforms. For example, MIL-101(Fe)-C_4_H_4_ was one of the MOFs used for controlled release of DOX by GSH/ATP-responsive stimulation.^21^ The DOX@MIL hybrid material was coated with poly(ethylene glycol-*co*-propylene glycol), F127, resulting in a material, F127@DOX@MIL, which releases DOX and Fe(III) ions through its decomposition under GSH and ATP stimulation. The released Fe(III) ions undergo a Fenton-like reaction with endogenous H_2_O_2_ and generate a toxic hydroxyl radical (^•^OH) that enhances the anticancer effect of DOX. DOX can also be encapsulated in the hybrid material prepared by coating iron-based MOF with hollow mesoporous organosilica nanoparticles (HMON) to maintain tumor remission during the postoperative period and prevent metastasis to the brain.^40^ Formulated as a nanosuspension, it can provide sustained release of the drug and increase the intracellular oxidative stress of tumors for remarkable tumor ferroptosis.

Abazari et al.^41^ developed a DOX delivery platform, DOX@DUT-32, using a Zn(II)-based MOF, Zn_4_O(BPDC)(BTCTB)_4/3_(DEF)_39.7_(H_2_O)_11.3_, known as DUT-32. They demonstrated that DOX release was 54% at physiological pH and nearly complete (98%) at pH 4.5. The new delivery system showed anticancer efficiency comparable to that of DOX alone, with minimal toxicity from the bare DUT-32. Wu et al.^42^ used ZIF-8 to develop a near-infrared (NIR) and pH-responsive DOX delivery nanoplatform, PDA-PCM@ZIF-8/DOX. The mild synthesis conditions allowed for a one-pot synthesis, achieving high loading (37.86%) and encapsulation efficiency (78.76%). ZIF-8 was modified with a thermally responsive phase-changing material, tetradecanol (PCM) for NIR-controlled release. Polydopamine (PDA) improved biocompatibility and served as a photothermal transfer agent, triggering the PCM′ thermal response. This dual-stimuli design resulted in a 78% drug release rate compared to just 21% without stimuli. PDA-PCM@ZIF-8/DOX demonstrated a high tumor inhibition rate *in vivo*, highlighting the effectiveness of the synergistic approach. The core–shell ZIF-8@Fe_3_O_4_ (ZIF: Zeolitic Imidazolate Frameworks, a group of MOFs that are topologically isomorphic with zeolites) stabilized with carboxymethylcellulose was also developed for an improved chemodynamic antitumor therapeutic strategy by promoting the Fenton reaction. Fe_3_O_4_ particles stabilized with carboxymethylcellulose target the lysosomes in cancer cells and generate more ^•^OH. On the other hand, this MOF-based drug delivery platform possessed minimized systemic toxicity. As in the previous case, ZIF-8 played a role in the storage of DOX and its pH-stimulating release.^22^

In addition to Fe- and Zn-based MOFs, Zr-based MOFs have also been investigated as potential carriers for DOX delivery. El-Bindary et al.^43^ synthesized a delivery platform by mixing the components of the Zr-MOF, ZrCl_4_, and 2-methylimidazole, with DOX in a one-pot synthesis and tested its anticancer activity on MCF-7 and HepG-2 cells. A drug release study at pH 7.4 and 5.0 using UV/vis spectroscopy showed a similar DOX release pattern to that of Fe-MOFs. Results indicated that DOX@Zr-MOF exhibited significant cytotoxicity, although bare DOX was slightly more effective. The antioxidative potentials of DOX, Zr-MOF, and DOX@Zr-MOF were also assessed, revealing that incorporating DOX into Zr-MOF reduces its antioxidative capacity, which may explain the lower cytotoxicity of DOX@Zr-MOF.

In a recent work,^23^ high control of DOX release and improved chemotherapeutic effect were achieved without additional functionalization of MOF, but by designing a well-defined MOF-on-MOF hybrid material in combination with photodynamic therapy (PDT). DOX was encapsulated in ZIF-8, and this material was assembled on the surface of Zr (IV)-based porphyrinic MOFs by using a MOF-on-MOF strategy, which enables a great degree of controllability in the thickness of ZIF-8 layer without requiring surface alterations on porphyrinic MOFs. The release of DOX was initiated by the degradation of ZIF-8 by the acidic microenvironment, while the role of Zr-porphyrin MOFs was to harvest light and convert tissue oxygen into reactive oxygen species (ROS) to kill tumor cells and thus enhance chemo/PDT therapy. Nano-ZIF-8 has also shown promise as a MOF for multimodal therapy, where more than one drug needs to be delivered to a specific site in a controlled manner. For example, ZIF-8 modified with the novel MCF-7 breast cancer targeting peptides (AR peptide) was used for simultaneous encapsulation of DOX and indocyanine green (ICG) with high encapsulation efficiency.^24^ Such a composite provides a dual targeting mechanism as the AR peptide is bound to it and ZIF-8 dissolves under acidic tumor conditions, allowing controlled release of ICG and DOX. It was shown that such a nontoxic multimodal platform is superior to ICG-activated PDT and PTT (Photothermal therapy) and DOX-based chemotherapy.^24^

**Figure 3:**
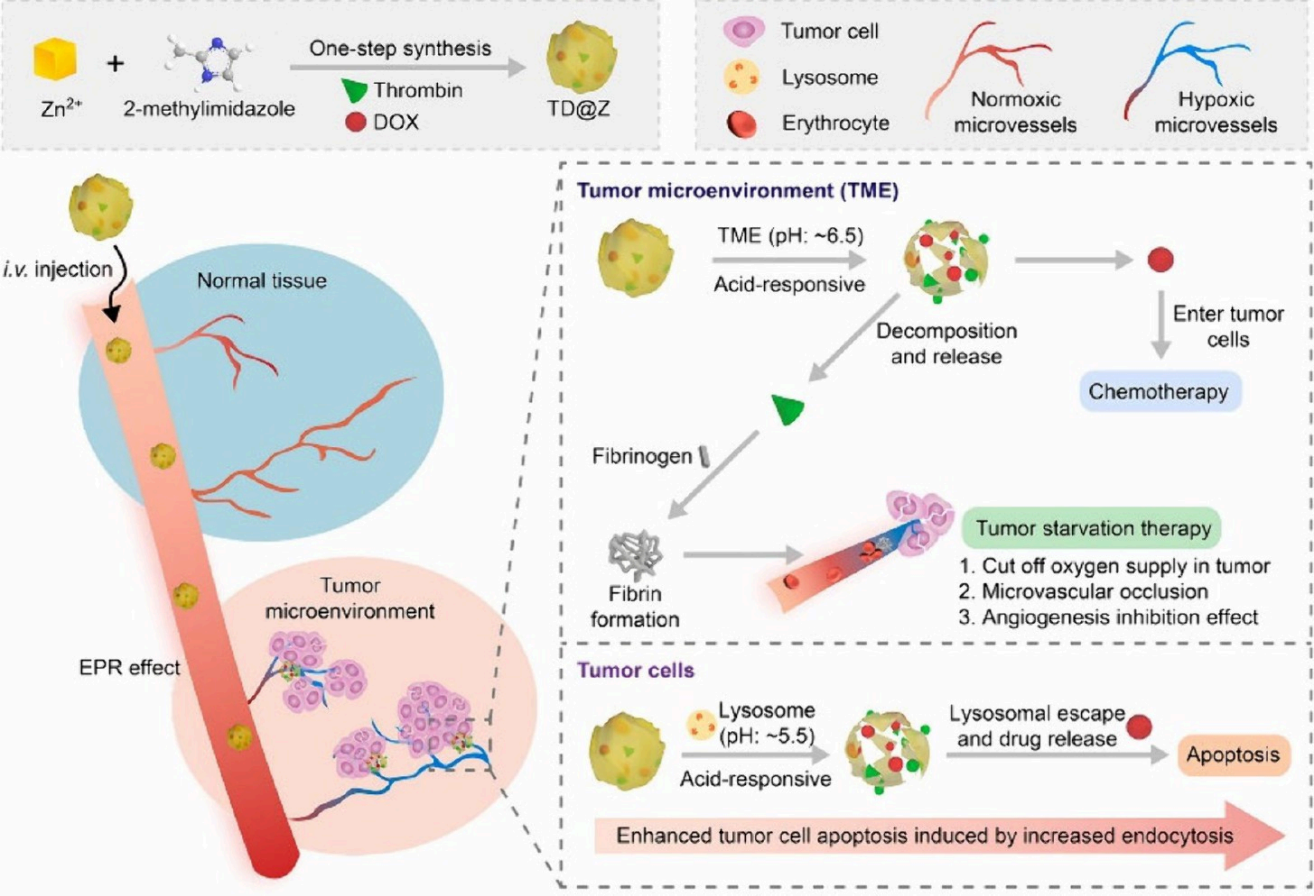
Synthesis pathway of TD@Z and its effect as tumor starvation therapy in the tumor microenvironment and chemotherapy effect in tumor cells. Adapted with permission from work by Liu et al.^25^ Copyright 2023, Elsevier.

In addition to chemotherapy, PTT and PDT, so-called “cancer starvation therapy”, are also frequently used, in which thrombin (a widely used endogenous coagulation protease) can be used to achieve vascular blockade of cancer cells. Similar to DOX, MOFs were shown to be effective when administered with thrombin, which is necessary due to the toxic effects of thrombin and the limited effects of cancer starvation therapy alone. Liu et al.^25^ used ZIF-8 to encapsulate thrombin and DOX to combine the microenvironment-triggered tumor starvation and chemotherapy simultaneously without the need for external stimuli as shown in Figure 3. The thrombin/DOX@ZIF-8, referred to as TD@Z and called “nanobomb”, showed a significant coagulation effect in the acidic cancer microenvironment and DOX release at the same time. The advantage of the “nanobomb” was reflected in the local accumulation of the drug and the enhanced chemotherapeutic effect of TD@Z through a restricted blood flow.

The drug delivery performance of MOFs for cancer treatment can be enhanced by combining them with microrobots, tiny mobile devices with promising biomedical potential. This unique tandem overcomes the limitations of each component individually. While MOFs improve the low drug-loading efficiency and poor degradability of microrobots, microrobots enhance the mobility and targeted drug delivery capabilities of MOFs. Terzopoulou et al.^44^ were the first to report MOF-based small-scale robots (MOFBOTs) with magnetic locomotion for DOX delivery and its targeted, controlled release through pH-responsive degradation of Fe@ZIF-8 MOF in cell cultures. ZIF-8 was chosen due to its biodegradability and minimal cytotoxicity upon degradation, while Fe provided magnetic properties that enabled magnetic navigation of microrobots. The structure of the MOFBOT (Fe@ZIF-8@GelMA) included biodegradable gelatin methacryloyl (GelMA), which degrades via enzymatic processes within cells. The authors first tested DOX release in buffers at pH 7.4 (physiological pH) and pH 6 (characteristic of tumor environments) over 96 h, demonstrating rapid release at pH 6 within the first 12 h. The cytotoxic effect of synthesized Fe@ZIF-8 and DOX-loaded Fe@ZIF-8 was also tested on the MDA-MB-231 breast cancer cell line. Results showed that bare Fe@ZIF-8 did not exhibit anticancer activity, maintaining a cell viability above 90%. In contrast, DOX-loaded Fe@ZIF-8 reduced cell viability to 35% after 72 h of treatment. The biocompatibility and biodegradability of magnetically active Fe@ZIF-8 were confirmed by using human embryonic kidney (HEK) cells. After 2 days of treatment with Fe@ZIF-8, cells could be attracted from suspension into a pellet by an external magnet. Confocal microscopy images revealed that Fe@ZIF-8 was localized within the cell membrane. The biodegradability of synthesized Fe@ZIF-8 was assessed by measuring the magnetic moment of cell pellets over time; after 48 h, the magnetic moment had decreased, and after 120 h, it disappeared completely, indicating the biodegradability of magnetic Fe@ZIF-8. Biocompatible and biodegradable magnetically active Fe@ZIF-8 was further integrated into a helical-shaped GelMA microchase, forming the MOFBOT. SH-SY5Y human neuroblastoma cells were selected to test the MOFBOT degradation due to their high activity of secreted proteases. Under experimental conditions, the MOFBOT was completely degraded after 14 days. These results provided a fully degradable magnetically maneuverable MOFBOT for drug delivery applications.

**Figure 4:**
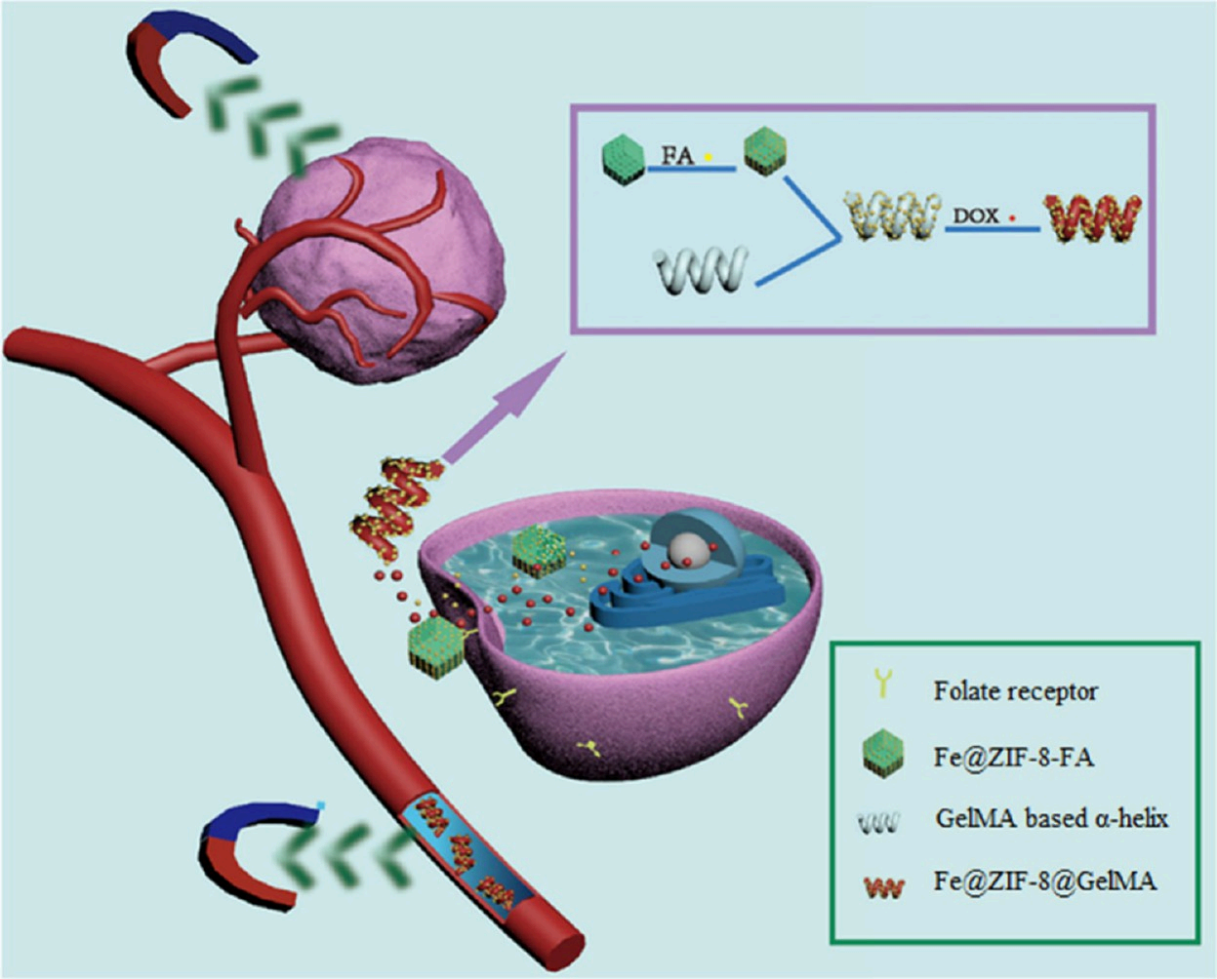
Illustration of magnetically controlled Fe@ZIF-8@GelMA microrobots for folate-targeted cancer therapy. Available under Creative Commons Attribution License 4.0 (CC BY 4.0). Adapted with permission from work by Ye et al.^45^ Copyright 2023, Cyborg and Bionic Systems.

An improved version of the MOFBOT was developed by Ye et al. in 2023.^45^ They enhanced the targeted delivery of DOX by additionally loading Fe@ZIF-8 with folic acid (FA) as shown in Figure 4. It is well-known that cancer cells often overexpress folate receptors (α-FA) on their surface; therefore, the authors proposed that incorporating FA into the previously synthesized MOFBOT could increase selectivity for cancer cells and improve DOX uptake via endocytosis. Indeed, when comparing the cytotoxic effect of the MOFBOT developed by Terzopoulou et al.^44^ to the FA-decorated MOFBOT developed by Wang et al., a significant improvement in the cytotoxic activity was observed, with cell inhibition rates increasing from 78% to 93%.

In 2024, Cao et al.^26^ constructed a slightly different magnetic MOF-based microrobot also as a pH-sensitive platform for DOX encapsulation by incorporating magnetically responsive components into biodegradable ZIF-8. The microrobots were organized in swarms whose shape and size can be easily controlled by adjusting the yaw (rotation) angle of the magnetic fields and the amount of nanoparticles, resulting in MNRs with a controllable size that can switch configurations instantly. Unlike the previous two cases, this MOF-based microrobot swarm eliminates the need for extra carriers, such as GelMA, and allows for a higher DOX loading. Similar release behavior under acidic conditions and cytotoxicity effects were observed. In vivo stability and antitumor activity of the microrobot swarm were demonstrated by studying premature release of DOX@MMRSs and the antitumor effect in mice with subcutaneous T24 tumors.

In addition to the commonly used Zn-, Zr-, and Fe-based MOFs, there are several studies on the potential applications of Cu- and Ag-based MOFs for the targeted release of DOX.^27,28^ For example, DOX was loaded onto CaO_2_ nanoparticles and encapsulated in a Cu-MOF and the surface was then modified with poly(ethylene)glycol (PEG), resulting in a CaO_2_-DOX-CuMOF/PEG hybrid material. A high GSH concentration triggers the decomposition of this material and promotes the release of DOX and CaO_2_, which favors the formation of H_2_O_2_ that generates ROS.^27^ On the other hand, an Ag-MOF was developed as a potential pH-responsive drug delivery platform for cancer treatment. Without additional coating with suitable polymers, Ag-MOF showed no DOX release at a physiological pH (7.4). However, at an acidic pH of 5.4, a gradual release of DOX was observed over 45 h.^28^ This platform also showed significant antibacterial activity against Gram-negative bacteria (*E. coli*).

Cyclodextrin-based metal–organic frameworks (CD-MOFs) make up an additional class of widely studied MOFs that have found applications in drug delivery for cancer treatment.^46,47^ These MOFs combine the cavity-like structure of cyclodextrins with the cage-like architecture typical of a MOF, enhancing the bioavailability and water solubility of active pharmaceutical compounds. Typically composed of eight glucose subunits (γ-CD) and alkali metal cations (Li^+^, Na^+^, K^+^, and R), CD-MOFs represent a highly suitable framework for biomedical applications. In addition, their advantage lies in environmentally friendly synthesis, which utilizes water and alcohol as solvents and their renewable nature. However, their practical application in drug delivery remains challenging due to CD-MOFs’ rapid decomposition and dissolution in physiological conditions.

In a recently published paper, Jia et al.^48^ developed a DOX delivery platform based on γ-CD-MOF by grafting graphene quantum dots (GQDs) onto its structure to provide strong fluorescence for tracking cell distribution. The platform was further coated with pH-responsive poly(ethylene glycol) dimethacrylate (PEGMA) to protect it from decomposition and ensure appropriate stimuli responsiveness. Enhanced targeting ability was achieved by immobilizing the AS1411 aptamer onto the hybrid material, PEGMA@GQDs@γ-CD-MOF, resulting in the new formulation named AS1411@PEGMA@GQDs@γ-CD-MOF. It was shown that this formulation possessed a higher DOX loading efficiency (89.1%) than the pristine γ-CD-MOF (45%) and GQDs@γ-CD-MOF (51.6%). Study of DOX release revealed that the release from AS1411@PEGMA@GQDs@γ-CD-MOF is strongly influenced by the pH of environment compared to γ-CD-MOF and GQDs@γ-CD-MOF. For DOX/γ-CD-MOF, drug release plateaued after 12 h, with 15.1% at pH 5.0 and 7.0% at pH 7.4. For DOX/GQDs@γ-CD-MOF, 50.2% of DOX was released at pH 5.0 and 36.3% at pH 7.4. Although the release improved after grafting GQDs into the MOF, it did not exhibit significant pH responsiveness. In contrast, DOX/AS1411@PEGMA@GQDs@γ-CD-MOF released 39.3% of DOX in 10 h at pH 5.0, and 98.0% after 4 days, showing a strong pH-responsive release. Additionally, the targeting specificity study showed that the DOX delivery system was effectively internalized through receptor-mediated endocytosis with high selectivity. *In vivo* antitumor studies in tumor-bearing mice demonstrated effective tumor suppression and partial ablation with minimal side effects. Therefore, the AS1411@PEGMA@GQD@γ-CD-MOF composite shows promise for effective DOX delivery and tumor growth inhibition, both *in vitro* and *in vivo*, with strong potential for anticancer therapy.

5-Fluorouracil (5-FU) is an anticancer drug with a significant antitumor activity. Although 5-FU has a significant cytotoxic effect, its main drawback is its rapid degradation (up to 10 min), which limits its broad clinical efficacy^49^ and necessitates the development of suitable carriers for this drug.^29−31^ Similar to DOX, MIL, ZIF, and CD series are mainly used for encapsulation of 5-FU and pH-dependent release of 5-FU, while the MOF surface was decorated with FC for targeted delivery.^29,50,51^ Xie et al.^30^ used MIL-101(Fe) MOF and the multifunctional nanosized MIL-101(Fe)@5-FU@FA (FA: folic acid) with a uniform particle size was utilized for drug delivery. It was found that MIL-101(Fe)@FU@FA effectively carries 5-FU to cancer cells that overexpress folate receptor, leading to an improvement in tumor therapy.

MOFs can also be considered as effective drug delivery platforms for the skin cancer treatment after encapsulation of 5-FU and sonidegib (SDG, therapeutic agent and targeting ligand).^31^ Such a hybrid material was incorporated into hyaluronic acid-hydroxypropyl methylcellulose gel, resulting in a topical gel formulation with improved skin deposition and reduced hyperplasia, nuclear pleomorphism, and dyskeratosis. Akbar et al.^29^ coated bimetallic (Fe and Co) MIL-88 with folic acid-conjugated chitosan and used it for targeted delivery of 5-FU with pH-response. The FC surface coating of 5-FU@bi-MIL88B-FC contributed to a slower release of 5-FU compared to bare 5-FU@bi-MIL88B, which is attributed to the gated effect phenomenon of FC. The release of 5-FU was twice as high under the pH conditions mimicking cancer cells. A chemodynamic agent with peroxidase-like activity, 5-FU@bi-MIL88B-FC, was created by adding Fe and Co to the MIL-88B structure.

Progress has also been made in the investigation of drug carrier systems for the brain. In this concept, studies are specifically focused on the development of effective treatments for neurodegenerative Alzheimer’s and Parkinson’s diseases. The therapies approved to date for Alzheimer’s disease (AD) are based on the use of acetylcholinesterase inhibitors or inhibitors of amyloid beta (Aβ) aggregation. In both cases, the most efficient therapy could be achieved by targeting drugs to the brain. For instance, a nanocarrier for huperzine A (Hup A) made of potassium CD-MOF was paired with microneedles made of dissolving hyaluronic acid to enhance medication transport to the brain through the nasal passages.^32^ Hup A, a reversible inhibitor of acetylcholinesterase, suffers from low solubility, and improving its uptake in the brain was achieved by encapsulating it in CD-MOF, which enhanced its water solubility. It was strengthened with stigmasterol and modified with lactoferrin. After intranasal administration, the hyaluronic acid-based microneedles dissolved rapidly and completely observed in the nasal mucosa, leaving CD-MOFs highly loaded with Hup A at the target site. This advanced drug delivery platform offers new opportunities to overcome the problem of rapid dissolution of nasal cilia and the barrier function of the nasal mucosa when delivering drugs from the nose to the brain.

The strategy of repurposing has shown promise, given the increasing need to develop effective treatments for these neurological disorders. Although metformin is primarily an oral antihyperglycemic agent for the treatment of type II diabetes, recent evidence shows that it can improve cognitive memory and reduce Aβ levels,^52,53^ while with appropriate encapsulation,^54,55^ the main challenges for its clinical application in the treatment of AD can be overcome. Vahed et al.^33^ constructed an alginate-coated nanosized ZIF-8 for effective metformin delivery under pH stimulation. The same group also developed an iron-based MOF delivery platform, (Fe)MIL-100-Met@alginate. Here, metformin was part of the MOF construction, while sodium alginate was used to coat the MOF surface and was responsible for pH-controlled release of metformin. This release system helped to improve the bioavailability and efficacy of metformin and reduce its side effects.

Parkinson’s disease (PD) is another neurological disorder where a MOF-based nasal administration strategy can further improve the efficiency of therapy. PD disease is characterized by a progressive degeneration of dopaminergic neurons in the brain, resulting in a significant loss of dopamine (DA) in the striatum.^56^ The most commonly used treatment to date is based on the administration of l-3,4-dihydroxyphenylalanine, which is known as l-dopa. However, l-dopa can also be metabolized in other body fluids, leading to side effects and discomfort in patients, while prolonged treatment is associated with dopamine dysregulation syndrome.^57^ To control DA release in the brain via noninvasive nasal administration,^58^ MOFs might be unique alternatives. For example, Pinna et al.^35^ were the first to incorporate dopamine into MOF composites (polymeric magnetic particles (PMPs), composed of Fe_3_O_4_ and polystyrene, forming PMP@MIL-88A systems) and test their potential for magnetophoretic applications. The role of PMP was to manipulate the movement of the carrier in the nasal passage to reach the olfactory nerve, which is directly connected to the central nervous system.^59^ Another advantage of the administration through the nose was the maximization of carrier size without the size restriction required in pharmaceutical delivery, thereby maximizing dopamine delivery to the target site.

In summary, thanks to various modification methods that have led to versatility, biocompatibility, and the ability to respond to external stimuli, MOF-based drug carriers hold great promise for various drug delivery applications, including cancer and neurological diseases, and represent advanced drug delivery systems that have the potential to revolutionize healthcare and improve patient outcomes.

## 3 MOFs as Sensing Platforms

The extended and functionalized surfaces of MOF materials enable them to capture and detect biomarkers even at extremely low concentrations, which has established MOFs as promising sensing materials for the development of ultrasensitive and highly selective sensors for disease diagnosis.^47^ To develop ultrasensitive biosensors, MOFs are often combined with biomolecules such as antibodies, enzymes, and nucleic acids. This is crucial for the early diagnosis of diseases such as cancer and AD, as the detection of low levels of biomarkers can significantly improve prognosis and treatment outcomes. Biosensors consist of a bioreceptor, a transducer component containing a semiconducting nanomaterial, and an electronic system. Thanks to the versatile properties of MOFs, which enable the integration of bioreceptors (such as enzymes, antibodies, cells, nucleic acids, or aptamers), and their nanoscale size, MOFs were successfully used as biosensors for the detection of cancer, AD biomarkers, live cancer cells, and microRNA.^60−63^ These MOF-based biosensors can recognize and bind biomarkers with high precision through the bioreceptor molecules embedded in the framework. The conversion of binding events into measurable signals using electrochemical (EC), electrochemical luminescent (ECL), fluorescent, and photoelectrochemical (PEC) methods facilitates an early and accurate diagnosis. For example, in a pioneering study of MOF-based biosensor, Zhu et al.^16^ synthesized a two-dimensional material (*N*,*N*′-bis(2-hydroxyethyl) dithiooxamidato-copper(II), commonly referred to as [Cu(H_2_dtoa)]), for the detection of HIV (Human immunodeficiency virus) and the blood coagulation enzyme thrombin. This sensor exhibited high sensitivity and selectivity by utilizing the principle of fluorescence quenching. Alizadeh et al.^64^ developed a highly sensitive ECL biosensor using mesoporous dca-Zr_12_ nanoplates to detect protein kinase activity, specifically protein kinase A (PKA). The biosensor utilized a unique interaction between phosphate groups and Zr^4+^ ions, where phosphorylation of peptides by PKA leads to the formation of phosphate sites that bind to the electrode surface, quenching the ECL signal. This “signal-off” strategy enables precise and rapid kinase detection. The biosensor demonstrated an impressive detection limit of 0.35 mU mL^–1^, with a successful application in drug-stimulated MCF-7 cell lysates. It also showed potential for screening kinase inhibitors. The use of MOFs such as Zr_12_ enhances ECL efficiency by stabilizing luminophores and minimizing intramolecular rotation. MOFs’ versatility in functionalization and stability makes them ideal carriers for developing sensitive analytical systems. This ECL biosensor platform presents a promising, simple, and sensitive method for monitoring kinase activity and could be applied in kinase-related research, diagnostics, and drug development, particularly in screening kinase inhibitors for disease treatment.

Biosensors that contain aptamers as biorecognition elements are called aptasensors, and in combination with electrochemical detection, they are often used for the specific detection of cancer cells.^65^ However, a disadvantage of electrochemical aptasensors is their lower sensitivity and stability compared to immunosensors, which are often the results of the wiring of the aptamers to the electrode. This decreases the amplification of the redox reaction generated by electrocatalysis or facilitates electron transfer to a lesser extent and impedes electron exchange at the aptasensor interface. MOFs were used to increase the surface area required for aptamer immobilization or to serve as a source of redox mediators that are collected or released during target binding.^63,66^ Recent advances in MOF-based biosensors for the treatment of cancer and neurological disorders are listed in Table 2. Bimetallic MOF-based aptasensors were successfully developed for the detection of cancer cells.^67−69^ For example, the carbohydrate antigen 125 aptamer was anchored on the MOFs consisting of Tb and Fe metal sites and used to detect MCF-17 cells.^67^ Aptamer strands were immobilized on carbon dot-doped Zr&Hf-MOF, and the resulting aptasensor was used as an ultrasensitive platform for the early diagnosis of HER2- and HER2-overexpressing living cancer cells.^74^

**Table 2:** Recent MOF-Based Sensor Studies for Cancer and Neurological Disorder Treatments^a^

	Sensor	MOF	Target	LOD	Detection	Ref
Cancer	Tb-MOF-on-Fe-MOF	TbFe-MOFs	CA125; MCF-7	58 μU/mL (CA125); 19 cells/mL (MCF-7)	EIS	(67)
	Zr-MOF@PO_4_-Apt	UiO-66–2NH_2_	MCF-7	31 cell/mL	EIS	(71)
	FA@UiO-66	UiO-66	HeLa	90 cells/mL	EIS	(72)
	antibody@ZIF-8/PDA–PEI	ZIF-8	PD-L1 antigen	0.035 pg/mL	ELISA	(73)
	Cr-MOF@CoPc	Cr-MOF	CT26	31 cells/mL (EIS);6 cells/mL (DPV)	EIS; DPV	(69)
	CDs@ZrHf-MOF	ZrHf MOFs	HER2	30 fg/mL (EIS); 19 fg/mL (DPV)	EIS; DPV	(74)
	CuBTC@MoS_2_-AuNPs	CuBTC (BTC: benzene-1,3,5-tricarboxylate)	CA125	0.0005 U/mL	DPV	(75)
	ssDNA@Mn-PCN-222	Mn-PCN-222	miRNA-21	0.4 fM	DPV	(76)
	Au NPs/Zr-MOF	Zr-MOF	miRNA-522	0.3 fM	ECL	(77)
	UiO-66-NH_2_@Ru	UiO-66-NH_2_ Ni-HAB	CA-153	0.0001 U/mL	ECL	(78)
	1T-MoS_2_@Ni-HAB					
	drDNA-BUT-88	BUT-88	miRNA-21; MUC-1	0.13 nM (miRNA-21); 4.50 nM (MUC-1)	FS	(79)
	Cu/UiO-66	UiO-66	CEA	0.01 ng/mL	FS	(80)
	Cu-MOF-NPs	Cu-MOF-NPs	AFP	1.18 ng/mL	FS	(81)
Alzheimer’s disease	‘[Ru(bpy)_3_^2+^/Zn-MOFs]; Au@NiFe MOFs	Zn-MOFs; NiFe MOFs	Amyloid β	13.8 fg/mL	ECL	(68)
	MIL101@Au–MoS_2_ QDs; Ru(bpy)_3_^2+^/NH_2_–UiO-66	MIL-101; NH_2_–UiO-66	Amyloid β	3.32 fg/mL	ECL	(82)
	Pd NPs@NH_2_-MIL-53	MIL-53	Amyloid β	3.4 fg/mL	ECL	(83)
	AuNPs/Fe-MIL-88-NH_2_	MIL-88	Aβ oligomers	71 fM	ECL	(84)
	Co-MOFs/ABEI	Co-MOFs	Amyloid β_42_	3 fg/mL	ECL	(85)
	g-C_3_N_4_ NS/Ru@ MOFs	Ru@MOFs	Amyloid β	3.9 fg/mL	ECL-RET	(66)
	Au NPs/Cu-MOFs	Cu-MOFs	Aβ oligomers	0.45 nM	DPV	(86)
	AuNPs@Cu-MOF-THS	Cu-MOF	Aβ oligomers	0.25 fM	DPV	(87)
	ThT@Er-MOF	Er-MOF ([Er(L)(DMF)_1.27_]_n_)	SSODN (part of the presenilin 1 gene); Aβ; ACh	0.517 pM (SSODN); 0.142 nM (Aβ); 0.492 nM (ACh);	FS	(88)
	CeONP-Res-PCM@ZIF-8/PDA	ZIF-8	Aβ oligomers	3.2 nM	FS	(89)
	Ru-MIL-101 (Al)-Apt-AuNPs/RecJF	MIL-101	Aβ oligomers	0.4 pg/mL	FS	(90)
	Cu–Al_2_O_3_-*g*-C_3_N_4_–Pd/UiO-66@PANI-MB	UiO-66	Amyloid β	3.3 fg/mL	SWV	(91)
	ZIF-8/Fer	ZIF-8	Aβ oligomers	0.5 μM (UV/vis); 10^–5^ μM (CV)	UV/vis; CV	(92)

a EIS: electrochemical impedance spectroscopy, DPV: differential pulse voltammetry, CV: cyclic voltammetry, ELISA: enzyme-linked immunosorbent assay, FS: fluorescence spectroscopy, UV/vis: UV/vis spectrophotometry, ECL: electrochemiluminescence, ECL-RET: electrochemiluminescence resonance energy transfer, SWV: square wave voltammetric methods.

To enhance the performance of immunosensors, Chen et al.^70^ developed a novel high-performance platform for the sensitive detection of AD-related biomarkers. The approach involved the use of a Zr-based MOF with peroxidase (POD)-like activity, which was designed to encapsulate the molecule methylene blue (MB). This MOF was then modified with a layer of gold nanoparticles, resulting in the creation of a nanozyme signal tag that could efficiently catalyze H_2_O_2_, producing hydroxyl radicals to amplify the signal. To further improve the binding efficiency of antibodies to target molecules, a DNA-aptamer-mediated strategy was employed, targeting the fragment crystallization region of antibodies (anti-Fc aptamer). This strategy was enhanced by incorporating a poly adenine sequence at the end of the aptamer, which promoted binding to the gold electrode while offering antifouling properties due to the hydrophilic nature of the phosphate group. This immunosensing platform was successfully applied to detect tau protein and BACE1, achieving detection limits of 3.34 fg mL^–1^ and 1.67 pg mL^–1^, respectively. Clinical analysis of 20 samples from volunteers of varying ages revealed significantly higher tau protein expression in the blood of elderly volunteers, indicating the potential of this strategy for early Alzheimer’s disease diagnosis.

It is important to note that in most MOF-based aptasensors, the immobilization of aptamers on the MOF is based on physical adsorption, π–π stacking, electrostatic interaction, or hydrogen bonding. This often leads to a loss of binding between the aptamer strands and the MOF substrate after the formation of a G-quadruplex between the DNA aptamer and the biomarker. This leads to a weakening of the detection performance of MOF-based biosensors. In a recent paper, Li et al.^71^ constructed a Zr-MOF-based multicomponent electrochemical aptasensor for breast cancer detection (MCF-7 cells).

**Figure 5:**
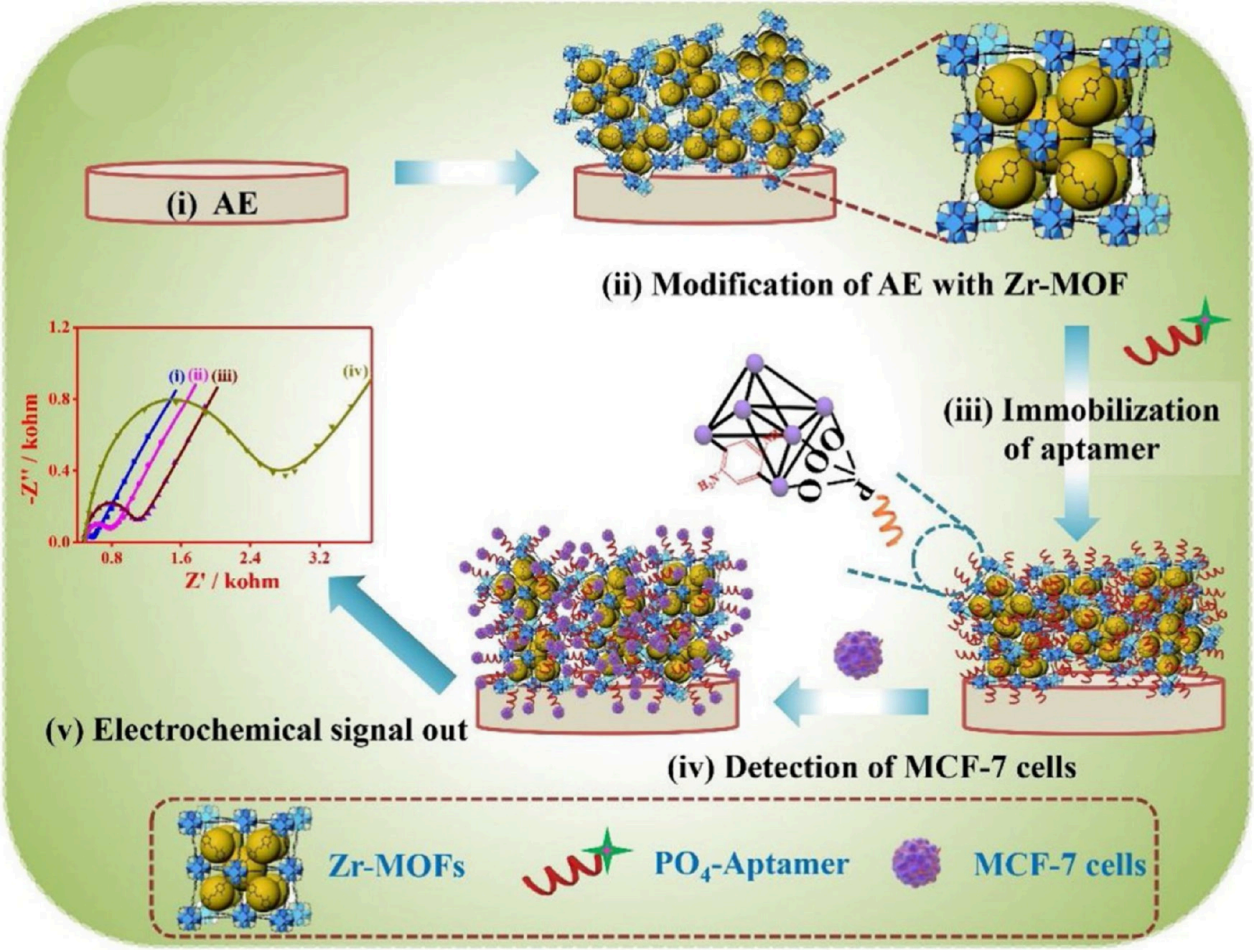
Fabrication of an improved Zr-MOF-based aptasensor for MCF-7 cell recognition using a UiO-66–2NH_2_-based sensor with PO_4_-modified aptamer strands. AE indicates the Au electrode. Adapted with permission from work by Li et al.^71^ Copyright 2020, Elsevier.

Figure 5 shows an aptasensor for MCF-7 cell recognition using a UiO-66–2NH_2_-based sensor with PO_4_-modified aptamer strands. The sensing abilities of aptasensors UiO-66, UiO-66-NH_2_, and UiO-66–2NH_2_ were compared, and UiO-66–2NH_2_ was reported to bind the aptamer significantly stronger via synergism of various interactions, resulting in improved stability in complex formation with MCF-7 cells and superior sensing ability compared to other aptasensors with extremely low detection limit. The detection limit of this aptasensor was 31 cells/mL in a wide range of MCF-7 cells, ranging from 100 to 100,000 cells/mL. The fabricated Zr-MOF-based biosensors were also found to be biocompatible upon testing their cytotoxicity on normal human cells (L929) and MCF-7 cells. A high survival rate of both cell types was achieved for all three types of Zr-MOFs based aptasensors, which was around 70% even at high concentrations of the biosensor (200 μg/mL).

In addition to aptamer-based MOF sensors, ELISA-MOF-based sensors (ELISA: Enzyme-Linked ImmunoSorbent Assays) sensors are also considered promising tools for cancer detection. Although ELISA belongs to the group of reliable tests for the detection of cancer biomarkers, it suffers from low sensitivity, a low limit of detection (LOD), and poor stability to temperature and pH changes in the environment. Zhand et al.^73^ pioneered the construction of an ELISA-MOF-based sensor for the detection of human PD-L1 antigen, a predictive cancer biomarker. By coating the ELISA plate with a thin protective film of ZIF-8 and polydopamine (PDA)–polyethylenimine (PEI), improved antibody immobilization was achieved. This immobilization contributed to a 225- and 15.12-fold increase in the LOD and sensitivity, respectively, compared to a commercial ELISA kit, and stability was maintained up to 55 °C in the pH range of 5–10.

Ma et al.^93^ evaluated four MOFs, Fe-MOF, Cu-MOF, Zn-MOF, and Zr-MOF, for cancer antigen 125 (CA125), a critical biomarker for early ovarian cancer diagnosis. Conventional CA125 detection relies on ELISA, and despite its effectiveness, it presents challenges related to complexity and time requirements. Cu-MOF showed the highest quenching efficiency (98%) and fluorescence differentiation, achieving a 30.5-fold increase upon CA125 binding, offering a simpler, faster alternative to ELISA. Cu-MOF’s effectiveness stemmed from its ability to release CA125-bound aptamers, unlike Fe-MOF, which inhibited release due to its positive charge. This research highlights Cu-MOF as a promising, efficient biosensor for early cancer detection, advancing MOF-based diagnostics with potential applications in clinical settings.

Given the clinical importance of alkaline phosphatase (ALP), a phosphomonoester hydrolase widely present in the human body and a biomarker for various diseases (such as secondary liver cancer and prostate cancer), developing practical methods for ALP detection is critical. Current ALP detection methods, such as electrochemistry, fluorescence, and chromatography, are typically reliant on complex, bulky equipment, lengthy detection times, and intricate procedures. This makes point-of-care testing (POCT) for ALP challenging, particularly in resource limited regions where simpler, rapid, and sensitive methods are needed. To address these limitations, Pan et al.^94^ conducted the analysis on the UiO-67 MOF-derived Fe–N–C nanozyme. The nanozyme’s activity was validated, showing that in the absence of ALP, 3,3′,5,5′-tetramethylbenzidine (TMB) was oxidized to produce a blue TMBox product. The system achieved a detection limit of 3.38 U L^–1^ for ALP, with recovery rates close to 100% for the tested ALP concentrations. This recovery rate confirms the accuracy and practicality of the Fe–N–C nanozyme approach. Additionally, a smartphone-based colorimetric assay allowed for visual detection of ALP, making it accessible for POCT in limited-resource settings. Overall, the Fe–N–C oxidase nanozyme and its visual detection capabilities offer a promising, user-friendly alternative for advancing diagnostic technology and improving patient care, especially in diverse and resource-limited healthcare environments.

MOFs may also contribute to the development of a green electrochemical biosensor, in which their role is to catalyze atom transfer radical polymerization (ATRP) for quantification of cancer miRNA instead of using traditional catalysts. Wang et al.^76^ developed such a biosensor for quantification of cancer miRNA-21 in serum, using PCN-222(Mn) (PCN: Porous Coordination Network). PCN-222(Mn) reacted with NH_2_-DNA by an amide reaction, and ATRP was initiated through specific recognition and host–guest reaction by miRNA-21. Ultrasensitive detection of miRNA-21 was achieved with a detection limit of 0.4 fM. The advantages of such a system compared to traditional methods are therefore not only shown in the lack of environmental impact but also in the improved efficiency of monomer polymerization, which leads to a higher sensitivity of the sensor.

Another example was developed by Dehnoei et al.:^95^ a dual biosensor based on the zirconium and praesidium based MOF (Zr/Pr MOF) for the rapid and ultrasensitive detection of miRNA-191, a biomarker linked to several health conditions. The Zr/Pr MOF exhibited activity like peroxidase and fluorescent features, enabling the oxidation of 3,3′,5,5′-tetramethylbenzidine (TMB) to blue oxTMB in the presence of H_2_O_2_. Label-free miRNA-191 binding caused significant changes in fluorescence and absorbance due to hybridization, which increased the peroxidase-like activity of the MOF. The system achieved a low detection limit of 0.69 pM for fluorescence and 8.62 pM for colorimetry. The approach is cost-effective, simple, and fast, making it a promising tool for biosensing and medical detection.

MOFs can also be functionalized with specific ligands or antibodies that selectively bind AD biomarkers. Similar to the detection of cancer biomarkers, the binding of ligands generates detectable signals through different mechanisms, including fluorescence, electrochemical responses, or colorimetric changes, enabling the use of MOFs as a sensor for the early diagnosis of AD. Some of the most important biomarkers for the early diagnosis of AD are Aβ peptides, which aggregate in the brain and form toxic plaques. The standard probe for Aβ detection is based on the Thioflavin-T (ThT) assay, in which enhanced fluorescence is observed after the interaction of the ThT dye with Aβ fibrils.^96^ Wang and co-workers^88^ developed a MOF-based fluorescent sensor for the smart detection of three AD biomarkers, including Aβ, presenilin 1, and acetylcholine (ACh), by incorporating this fluorescent dye into a MOF structure with erbium metal sites (Er-MOF). The presence of the fluorescent dye ThT and the luminescent Er in this probe lead the dual-emission ThT@Er-MOF sensor to be fabricated. This probe detects Aβ with excellent sensitivity and high selectivity, achieving a low LOD of 0.142 nM and a wide linear range of 0–40 nM over Aβ. This sensor was reported to detect not only Aβ but also presenilin 1 (a gene sequence essential for AD) and ACh in cerebrospinal fluid with high sensitivity, wide detection range, and low LOD values.

ZIF-8 was also utilized in the construction of sensors for Aβ detection. .For example, Qin et al.^92^ prepared ferrocene-encapsulated ZIF-8 and used it for the indirect dual detection of Aβ oligomers. This sensor relies on the strong interaction between Zn^2+^ ions and Aβ, resulting in the release of ferrocene. Ferrocene provides significant optical and electrochemical signals that can be detected by UV/vis spectroscopy and CV. The dual determination of Aβ oligomers contributes to rapid qualitative and precise quantitative analyses over a wide detection range of 10^–5^ to 10^2^ μM and shows good feasibility in artificial cerebrospinal fluid. These results suggest that ZIF-8@Fer could be an important analytical sensor for the *in vivo* detection of Aβ-oligomers. Yan et al.^89^ also used ZIF-8 in combination with PDA, resveratrol (Res), ceria nanoparticles (CeO_2_ NPs), and tetradecanol as a phase change material (PCM) to develop fluorescent CeO_2_ NP-Res-PCM@ZIF-8/PDA/Apt sensors for Aβ. The synergistic effect of PDA, CeO_2_ NPs, and Res in one system resulted in multifunctional effects of this complex nanocomposite, reflected in its sensory and therapeutic abilities to inhibit Aβ aggregation, degrade Aβ fibrils, and alleviate Aβ-induced oxidative stress and neuronal apoptosis. Here, PDA was chosen for its strong NIR absorption (NIR: Near Infrared), making it suitable as a photothermal agent with high efficiency conversion for photothermal therapy, and thanks to its high affinity for interacting with various biomolecules.^97^ Res was selected for its anti-AD potential and PCM was used to control Res release through NIR. The role of CeO_2_ NPs was to scavenge ROS thanks to their strong and recyclable ROS scavenger ability, which originates from the loss of electron by Ce(III) to form Ce(IV).^98^

**Figure 6:**
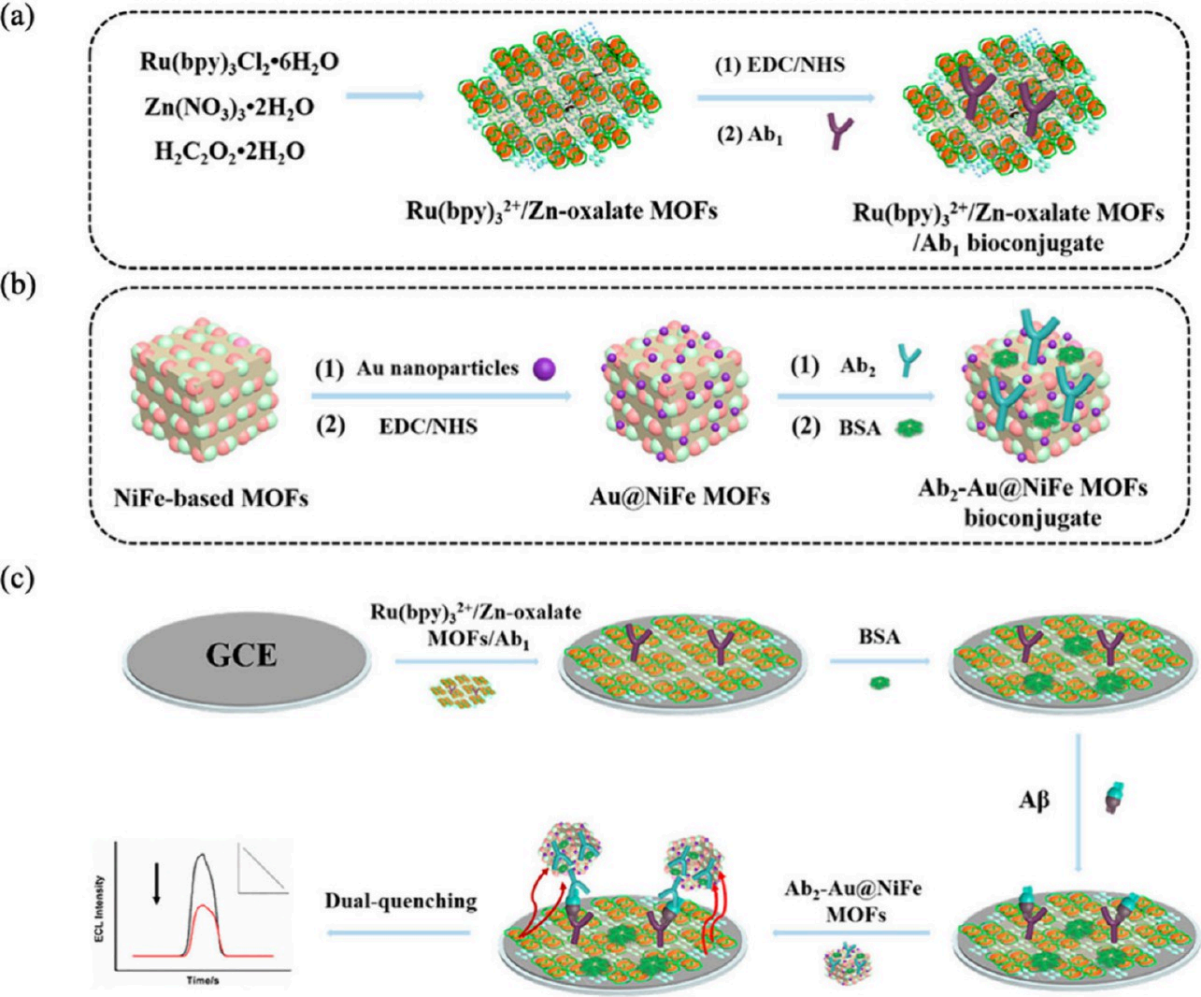
(a) Preparation of Ru(bpy)_3_^2+^/Zn-oxalate MOFs/Ab1 bioconjugate; (b) Ab_2_-Au@NiFeMOFs bioconjugate; (c) Construction process of the proposed ECL immunosensor.^68^ Copyright 2019, American Chemical Society.

Zhao et al.^68^ developed a dual-quenching MOF based immunosensor for the highly sensitive detection of Aβ. This sensor is based on two hybrid MOFs as shown in Figure 6 where the first consists of encapsulated Ru(bpy)_3_^2+^ in a Zn-MOF, which acts as a luminophore, and the second structure comprises Au@NiFe MOFs, which serve as a dual quencher owing to the ECL acceptor properties of both the AuNPs and the NiFe-based MOFs. The role of porous Au@NiFe MOFs was to quench the fluorescence of Ru(bpy)_3_^2+^/Zn-MOF through the FRET (Foster Resonance Energy Transfer) process, thereby significantly improving the sensitivity of the immunosensor. The ECL immunosensor showed excellent selectivity, stability, and reproducibility, demonstrating a promising quenching strategy for Aβ detection.

Dong et al.^82^ also used Ru(bpy)_3_^2+^ to construct a dual MOF immunosensor for the detection of Aβ. Their sensors consisted of MIL-101 and NH_2_–UiO-66, with Ru(bpy)_3_^2+^ inserted into NH_2_–UiO-66 and MoS_2_ QDs linked to MIL-101 via Au–S bonds, which served as markers for secondary antibodies (Ab2). The Ru(bpy)_3_^2+^/NH_2_–UiO-66 formed possess a large surface area for adsorption of antibodies and exhibits significant luminescence, which can be quenched by MIL-101QAu-MoS_2_ QDs. The amount of Ab2-MIL-101@Au–MoS_2_ QDs bound to the antigen gradually increases with Aβ concentration, resulting in a “signal-off” response for an accurate estimation of Aβ. The obtained sensor has a wide linear range from 1 × 10^–5^ up to 50 ng/mL and a detection limit of 3.32 fg/mL for Aβ. Research has consistently shown that MOFs are excellent materials for creating AD biosensors that are both sensitive and selective.

Besides Aβ peptides, another important biomarker for the diagnosis of AD is acetylcholine (ATCh). ATCh deficiency plays a crucial role in AD, making acetylcholinesterase (AChE), the enzyme that hydrolyzes ATCh, a key target for AD therapy. Traditional detection methods for AChE activity, such as electrochemical- and fluorescence-based techniques, are complex and require large instruments, underscoring the need for simpler, more efficient detection technologies. Nanozymes, synthetic materials that mimic the catalytic activity of natural enzymes, have gained attention for their stability, cost-effectiveness, and performance. MOF-based nanozymes, particularly those doped with noble metals, offer significant advantages due to their high surface area and stability. The study of Bai et al.^99^ focused on the development of a novel MOF-based nanozyme, PCN-224-Pt, by incorporating platinum nanoparticles (Pt NPs) onto a Zr-MOF (PCN-224) using an *in situ* reduction strategy. The resulting nanozyme exhibited strong peroxidase-like (POD) activity and was used to create a highly selective colorimetric platform for monitoring the AChE activity and its inhibitors. The detection principle relies on AChE catalyzing the hydrolysis of ATCh into TCh (thiocholine), which inhibits the oxidation of 3,3′,5,5′-tetramethylbenzidine (TMB) into the colored product ox-TMB. In the presence of an AChE inhibitor, this pathway is blocked, allowing TMB to be oxidized into ox-TMB under the catalysis of PCN-224-Pt. The platform showed excellent sensitivity, with a detection range of 3.125–100 mU mL^–1^ for AChE activity and a LOD as low as 2 mU mL^–1^, while also detecting AChE inhibitors with an LOD of 0.49 ngmL^–1^. This innovative approach provides a rapid, sensitive, and selective method for screening AChE activity and its inhibitors, offering significant advantages over conventional detection methods with promising applications in diagnostics and therapeutics.

MOFs can also be modified to detect key factors that contribute to the development of PD. PD is marked by the accumulation of pathological α-synuclein (α-Syn) and the degeneration of dopaminergic neurons, may be detected early through noninvasive methods.^100^ α-Syn is initially detected in the gut before it affects the brain, presenting a unique opportunity for early monitoring through oral bioprobe delivery. This method offers a user-friendly, noninvasive approach for detecting PD; however, challenges arise due to the instability of probes in the acidic environment of the gastrointestinal (GI) tract. To overcome this, luminescent terbium-based metal–organic frameworks (Tb-MOFs) have been proposed as robust carriers for aptamers, which remain stable under harsh GI conditions.^100^ These MOFs have precise pore sizes that effectively protect and enhance the stability of aptamers, ensuring that they can function as reliable biosensors for PD detection. The Tb-based luminescent MOF system, when combined with Pt nanoparticles and aptamers, enables noninvasive monitoring of PD by detecting fluorescence changes in response to the presence of α-Syn. When α-Syn is encountered in the gut, the aptamers within the Tb-MOF probe bind to it, forming a Pt-Aptamer/α-Syn complex that triggers a fluorescence “turn-on” signal, signaling the presence of α-Syn. Additionally, the integrity of the Tb-MOF probe remains stable throughout the GI tract, allowing it to be excreted in the feces, where the fluorescence intensity can be measured for diagnosis. This approach demonstrates significant potential as a safe, effective, and early stage monitoring method for PD, paving the way for improved diagnostic strategies. The ability to detect α-Syn noninvasively through the oral administration of Tb-based luminescent MOF probes opens a new avenue for early diagnosis and management of PD, offering a more accessible and efficient alternative to traditional diagnostic methods.

Overall, all these recent studies highlight that MOFs can be used in various detection systems, and the possibility of their easy modification for efficient performance in capturing different biomolecules distinguishes them from conventional materials such as AuNPs, carbon nanotubes (CNTs), quantum dots (QDs), graphene, and magnetic nanobeads.^101−106^

## 4 Computational Studies on Biomedical Applications of MOFs

We so far reviewed the pioneering experimental studies for the biomedical applications of MOFs. Computational modeling, specifically quantum mechanics (QM) calculations and molecular simulations, have been very useful to better understand the potential of MOFs in biomedical processes by revealing the molecular interactions between the guest molecules and MOF structures. For example, density functional theory (DFT) calculations have been widely used to describe specific interactions between drug molecules and MOFs and to identify the most favorable adsorption sites in the structures.^107,108^ Grand canonical Monte Carlo (GCMC) and configurational bias Monte Carlo (CBMC) simulations have been used for predicting saturated drug loading capacities of MOFs, whereas molecular dynamics (MD) simulations have been widely used to examine diffusion of drug molecules inside the pores of MOFs. Technical details of these methods can be found in the literature.^109−111^

### 4.1 Drug Storage and Delivery

**Figure 7:**
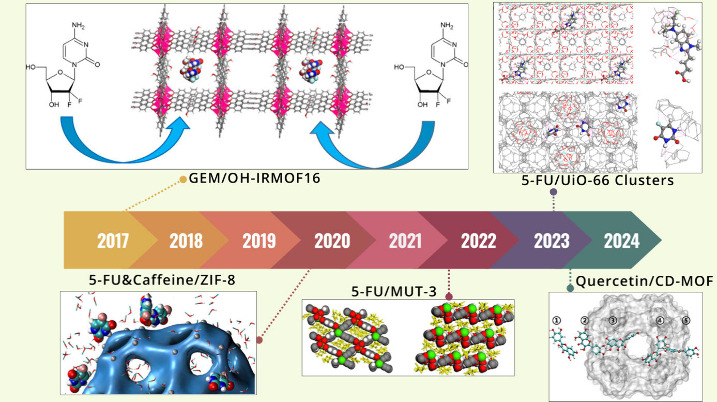
Illustrative timeline of the computational studies for drug storage and delivery using various MOF structures. Top: GEM drug loading in OH-IRMOF-16, and 5-FU drug storage in UiO-66-COOH and UiO-66-NDC unit cell (adapted with permission^112,113^ Copyright 2017, Elsevier; Copyright 2023, Wiley). Bottom: 5-FU and caffeine drug storage in ZIF-8 structures, 5-FU drug storage in MUT-3 structure, and quercetin in CD-MOF (adapted with permission^113−116^ Copyright 2020, American Chemical Society; Copyright 2022, American Chemical Society; Copyright 2024, Elsevier).

The design, understanding, and optimization of MOFs for drug storage and delivery have been greatly advanced by computational studies in the past few years. The timeline showing the computational studies on drug storage and delivery using MOFs is given in Figure 7. The first computational study^108^ in this field focused on the interaction between ibuprofen, an analgesic and anti-inflammatory drug, and two mesoporous MOFs, MIL-101 and UMCM-1. Results of DFT, MC, and MD studies showed that the highest amount of ibuprofen in MIL-101 is 1.11 g/g, in good agreement with the loading that was reported experimentally, 1.37 g/g. Results also showed that MIL-101 has four-times higher ibuprofen loading capacity than the mesoporous silica MCM-41. Therefore, a small amount of MIL-101 can be adequate for large dosages.

Table 3 lists the computational studies focusing on drug storage and delivery of different types of MOFs. For example, Kotzabasaki and co-workers^117^ investigated the adsorption of an anticancer drug (GEM: gemcitabine) in IRMOF-74(III) and its OH-functionalized form. The maximum GEM loading in these two MOFs was ∼1.13 g/g, which was much higher compared to lipid-coated mesoporous silica nanoparticles (0.4 g/g) and quite lower compared to liposomes (1.5 g/g).^117^ The diffusion coefficients of GEM molecules were also reported in the range of 8 × 10^–7^–12.5 × 10^–8^ cm^2^/s, indicating slow release of GEM molecules from IRMOF-74(III) and its OH-functionalized form. The same research group also computed the saturated GEM loading in OH-functionalized IRMOF-16 materials as 4.343 g/g.^112^ A slower GEM release (almost half the time for total release) was reported for the OH-functionalized IRMOF-16. To analyze the positioning of GEM molecules within MOF pores during MD simulations, results showed that GEM’s amino group primarily interacts with the corners of IRMOF-74-III, allowing the rest of the molecule to rotate freely and form occasional hydrogen bonds.

**Table 3:** Computational Studies of MOFs for Drug Storage and Delivery Applications^a^

MOF	Drug	Method	Remark	Ref
MIL-47	Ibuprofen	GCMC	Separation of racemic mixtures of ibuprofen by chiral HMOF-1, scalemic mixtures by nonchiral MIL-47 and MIL-53 structures.	(118)
MIL-53				
HMOF-1	Lysine			
MIL-53	Ibuprofen	GCMC	MIL-53 and MIL-101 align well with experimental ibuprofen adsorption data, BioMOF-100 exhibits outstanding capacity (1969 mg g^–1^) due to DMA cations, and CDMOF-1 shows potential for controlled drug release.	(119)
CD-MOF-1				
MOF-74				
MIL-100				
MIL-101				
BioMOF-100				
UiO-66	Taxol	MD	Understanding adsorption sites.	(120)
UiO-67	Cisplatin			
MOF-74	MTX	CBMC	High MTX (2.78 g/g) and 5-FU (4.24 g/g) loading in RAVXIX compared to mesoporous silica materials, molecular sieves, PLGA-based micelles, magnetic nanocomposites.	(121)
	5-FU	MD		
IRMOF-74-III	GEM	GCMC	Similar GEM loading capacities to liposomes (1.5 g/g), slow GEM diffusion (∼10^–7^ cm^2^/s).	(117)
OH-IRMOF-74-III		MD		
IRMOF-16	GEM	GCMC	High GEM loading capacity (4.3 g/g) outperforming more than ten times lipid coated mesoporous silica particles.	(112)
PI-3-COF	5-FU	DFT	Slow 5-FU diffusion (1.48 × 10^–7^ cm^2^/s).	(122)
		MD		
MPF	6-mercaptopurine	MD	Drug diffusivities change (0.12–0.26 × 10^–5^ cm^2^/s) under external electric field ranging from 0.25 to 1 V/nm.	(123)
ZIF-8P (without surface)	5-FU	GCMC	ZIF-8S model provides a good agreement with experiment.	(114)
ZIF-8S (with surface)	Caffeine			
UiO-66	DOX	GCMC	Understanding adsorption sites.	(124)
MIL-101(Cr)				
ZIF-8	Gabapentin Levetiracetam Phenytoin Valproate	CBMC	The highest drug loading in ZIF-8.	(125)
ZIF-67				
ZIF-90		MD		
UiO-AZB	5-FU	GCMC	Solvent effects on drug adsorption.	(126)
CuBTC	IBU			
NH_2_-MIL-53(Al)	HU			
MUT-2	5-FU	GCMC	Higher 5-FU drug loading (0.8 g/g) of MUT-2 compared to that of (0.5 g/g) MOF-123.	(127)
MUT-3	5-FU	GCMC	Comparable 5-FU drug loading (0.7 g/g) of MUT-3 with MUT-2.	(115)
UiO-66	Temozolomide	MD	The interaction energies showed that the interaction of temazolomide and alendronate with UiO-66 is more favorable than 5-FU with UiO-66.	(128)
	Alendronate			
	5-FU			
ZIF-8S	Cytarabine	GCMC	ZIF-8S model accurately described the adsorption sites.	(129)
UiO-66	Bendamustine	GCMC	COOH functionalized UiO-66 has the highest bendamustine loading of 20.94 wt %. NDC functionalized UiO-66 has the highest 5-FU loading of 51.23 wt %.	(113)
UiO-66-COOH				
UiO-66-NDC				
UiO-66-NH_2_	5-FU			
UiO-67				
MUT-8	Quercetin	GCMC	Saturated quercetin loading at extremely low fugacity.	(130)
CD-MOF	Quercetin	MD	Quercetin spontaneously adsorbs on CD-MOF verified by Gibbs free energy −4.4 kcal/mol.	(116)

a HMOF: heterometal–organic framework, GCMC: Grand canonical Monte Carlo, MD: molecular dynamics, CBMC: configurational bias Monte Carlo, MTX: methotrexate, 5-FU: 5-fluorouracil, IRMOF: Isoreticular MOF, GEM: gemcitabine, COF: covalent organic framework, MPF: peptide-based MOF.

Bueno-Perez et al.^118^ studied ibuprofen and an essential amino acid (l-lysine) adsorption in MIL-47(V), MIL-53(Cr), and HMOF-1 (heterometal–organic framework), which are modeled as rigid, to save computational time, using GCMC simulations. They reported that the chiral structure HMOF-1 can separate racemic ibuprofen mixture, whereas nonchiral structures (MIL-47 and MIL-53) separated scalemic ibuprofen mixture. This study showed how molecular simulations can be used to predict molecular adsorption and enantioselectivity in porous materials and provided insights into the confinement of the ibuprofen molecules in narrow pores of MIL-47 and MIL-53 and chiral environments within the MOF structures. Bernini et al.^119^ examined ibuprofen uptake in 6 different MOFs, namely MIL-53, CD-MOF-1 (CD = cyclodextrin), MOF-74, MIL-100, MIL-101, and BioMOF-100. Bio-MOF-100 exhibited the maximum ibuprofen uptake (1.975 g/g) thanks to its large pore volume (2.9 cm^3^/g). The ibuprofen uptake capacity of Bio-MOF-100 was reported to be six-times higher than the value found in mesoporous silicas.

**Figure 8:**
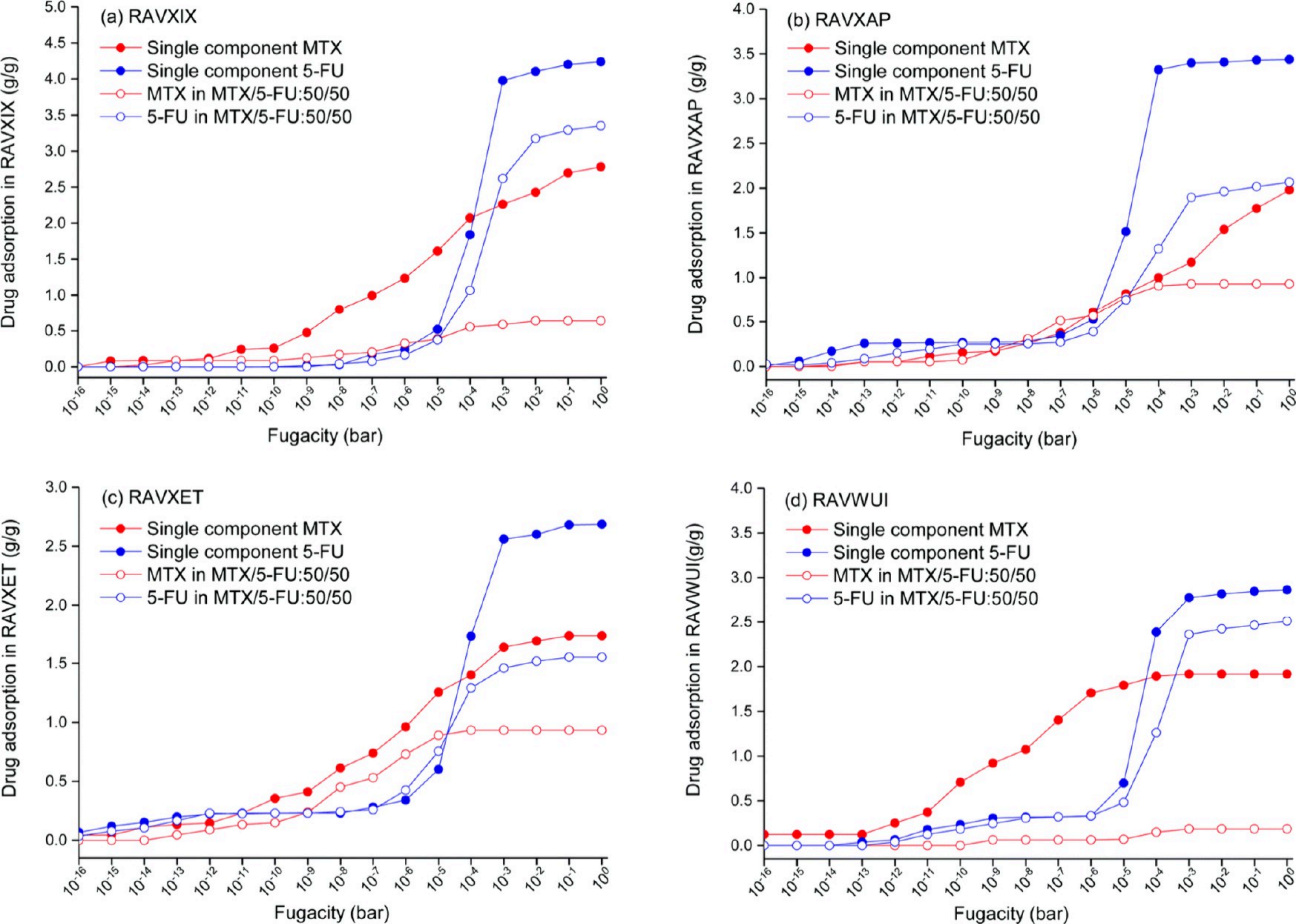
Single-component adsorption isotherms of MTX and 5-FU with their mixture adsorption isotherms in (a) RAVXIX, (b) RAVXAP, (c) RAVXET, and (d) RAVWUI.^121^ Copyright 2017, Royal Society of Chemistry.

Co-adsorption of anticancer drug molecules, MTX, and 5-FU were studied in MOF-74 series by Erucar et al.^121^ Results showed that single-component drug adsorption can differ significantly from mixture adsorption due to competition between drug molecules for the same adsorption sites on the MOF, potentially reducing drug uptake in mixtures, as compared to single-component cases, illustrated in Figure 8 through a comparison of their respective adsorption isotherms. For strongly adsorbed MTX, the decrease is more apparent. At 1 bar, the overall amount of mixture drug uptakes of 2.5, 2.7, 3.0, and 4.0 g/g is less than the total amount of single-component MTX and 5-FU uptakes of 4.4, 4.8, 5.4, and 7.0 g/g in RAVXET, RAVWUI, RAVXAP, and RAVXIX, respectively. The names of these MOFs are reported based on their refcodes, which are unique six-character identifiers assigned by the Cambridge Crystallographic Data Center (CCDC).^131^ In order to study a representative material set having a range of structural parameters, such as pore diameters, porosities, and surface areas, 10 MOFs from the MOF-74 series were examined. Except for RAVWUI, which contains Zn, all of the MOF-74 compounds contain Mg metal. The results of CBMC simulations showed that among 10 different MOF-74 materials, RAVXIX (known as IRMOF-74-IX) was identified as the most promising structure for MTX (2.8 g/g) and 5-FU (4.2 g/g) uptake thanks to its large pore volume (3.74 cm^3^/g) and pore size. This computational study helps researchers to understand multidrug delivery systems using MOF carriers.

Hashemzadeh et al.^122^ examined both adsorption and diffusion of 5-FU in PI-3-COF (PI: Polyimide, COF: Covalent Organic Framework) to develop a novel drug delivery system. The preferential sitting of 5-FU molecules was mostly found on the PI-3-COF surface because of the hydrogen bond and π–π interactions. This framework might be useful for the controlled 5-FU drug release thanks to its low self-diffusivity (1.48 × 10^–7^ cm^2^/s). Li et al.^113^ examined the storage of two anticancer drugs, 5-FU and bendamustine, in five MOFs (UiO-66, UiO-66-NH_2_, UiO-66-COOH, UiO-67, and UiO-66-NDC; NDC= 1,4-naphthalenedicarboxylic acid) and reported that UiO-66-COOH is a promising candidate with a high bendamustine loading capacity (20.94 wt %) thanks to the interaction between carboxyl groups of the framework and negatively charged N atoms of the drug. UiO-66-NDC exhibited the highest 5-FU uptake as 51.23 wt % with its large pore aperture (7 Å).

Alavijeh and co-workers investigated the potential of two different MOFs, MUT-2^127^ and MUT-3,^115^ for 5-FU encapsulation and reported their 5-FU uptakes as 0.841 and 0.717 g/g, respectively, outperforming zeolite ZSM-5 (0.38 g/g)^132^ and cross-linked microspheres (0.15 g/g).^133^ Sun et al.^125^ investigated the uptake of several antiepileptic drugs including valproate, gabapentin, levetiracetam, and phenytoin in three MOFs, ZIF-8, ZIF-90, and ZIF-67. Among these MOFs, ZIF-8 exhibited the maximum drug uptake capacity of 8.5, 9.94, 9.52, and 12.01 wt % for these drugs at 298 K, respectively, indicating that this framework has significant potential as antiepileptic drug carrier. Boroushaki et al.^128^ and Filippousi et al.^120^ performed MD simulations to investigate the delivery of anticancer drugs (5-FU, paclitaxel and cisplatin) in UiO-MOF series and concluded that the interactions between drug molecules and MOF structures determine the drug release, as the microencapsulated formulations are responsible for the controlled release rates. Filippousi et al.^120^ also showed that drug-loaded UiO-MOFs demonstrated superior anticancer activity in cytotoxicity tests for both cell lines when compared to free paclitaxel and cisplatin solutions at varying doses. Froudakis’s group^130^ performed GCMC simulations to compute quercetin, an anticancer drug, loading capacity (0.3 g/g) of a Mg-based MUT-8 material at 10 bar and 310 K. They noted that quercetin adsorption in MUT-8 reaches saturation even at extremely low-pressure range (∼1 Pa). This results from the strong interaction between the drug molecules and framework of MUT-8, as well as the advantageous pore size of MUT-8 that makes it easier for drug molecules to be accommodated.

Shahabi et al.^123^ performed MD simulations to compute the self-diffusion coefficient of an anticancer and immunosuppressive drug, 6-mercaptopurine in a peptide-based MOF (MPF) under different electrical fields. They demonstrated that a drug can be released more rapidly from a porous MOF structure when subjected to an electric field ranging from 0.25 to 1 V/nm, with self-diffusion coefficients of 0.12–0.26 × 10^–5^ cm^2^/s. This study shows that modifying the diffusion coefficient of drug molecules within a MOF structure using an electric field could have significant implications for controlled drug delivery.

Validating the results of computational studies by combining them with experiments is very important to further developing the use of MOFs in real biomedical applications. In this regard, Markopoulou et al.^124^ performed GCMC simulations to compute DOX loading in MIL-101(Cr) and UiO-66 to validate their experimental DOX uptake results. Simulations for MIL-101(Cr) showed a theoretical maximum loading of 1.16 g/g, ten-times higher than the experimental results, confirming pore adsorption of DOX in MIL-101(Cr). On the other hand, simulations revealed a DOX adsorption of zero for UiO-66, indicating the loading of the drug on the surface. Proenza et al.^114^ investigated the adsorption and release of 5-FU and caffeine in and from ZIF-8 by utilizing Gibbs-ensemble Monte Carlo approach considering two models for ZIF-8; one without surface (ZIF-8P) and one with surface (ZIF-8S). The guest molecules were unable to enter ZIF-8S’s interior pores, while solvents like methanol or water could. The lack of surface and solvent effects in ZIF-8P model caused a poor quantitative agreement with the experimental results.^134^ Singh et al.^129^ computationally and experimentally investigated the adsorption behavior of a chemotherapy drug (cytarabine) in the same defected ZIF-8 structures. At 298 K and 1 bar, cytarabine uptakes in ZIF-8P and ZIF-8S were calculated as 9.5 and 33.4 wt %, respectively. In the ZIF-8S model, drug loading was 30.5 wt % in the presence of solvent since the solvent molecule occupied the free volume of ZIF-8. The ZIF-8S model accurately described the adsorption sites, allowing uncoordinated zinc ions to interact with drug molecules by altering the periodic conditions from an atomistic level to a higher molecular level. We note that ZIF-8S is found to be a more realistic model, resulting in good agreement with experimental cytarabine loading.

Sose and co-workers^126^ performed GCMC simulations to examine the importance of the presence of solvent (ethanol) molecules during adsorption of three drugs (5-FU, ibuprofen and hydroxyurea) in biocompatible MOFs, UiO-AZB (AZB = azobenzene), CuBTC, and NH_2_-MIL-53(Al). Among these materials, UiO-AZB exhibited the highest drug uptake in the presence of ethanol thanks to its larger interior pore sizes (13.7 Å). They also reported that the drug adsorption was driven by electrostatic interactions in both the presence and absence of the solvent condition at lower pressures. These studies demonstrate that including solvent molecules in molecular simulations may affect drug uptake in MOFs.

### 4.2 Binding Energy

Binding energy, which indicates how strong and stable a guest–MOF complex can be, could help us to better understand the ability of MOFs to encapsulate and release certain substrates, which is crucial for optimizing their performance in medical treatments. For example, DFT calculations were performed to understand the interactions between oseltamivir, a potential drug for the treatment of COVID-19, and a novel yttrium MOF (Y-MOF).^135^ The linker of the Y-MOF has a large aromatic backbone that can strongly interact with DNA sequences through π–π stacking interactions, which establishes the idea of using DNA-functionalized MOFs to create a unique sensor. DFT calculations showed that oseltamivir interacts preferentially with Y-metal sites via its carbonyl group. This is the first example of how selective detection of the COVID-19 biomarker can be achieved using a MOF via interaction mechanisms such as hydrogen bonding and π–π stacking. In a recent study, Zhao et al.^116^ performed molecular docking simulations to better understand the binding mechanism between quercetin molecules and CD-MOF framework. The computed low free energy (−4.4 kcal/mol) was attributed to weak interactions and spontaneous adsorption of quercetin molecules onto γ-CD.

### 4.3 Biosensors

**Figure 9:**
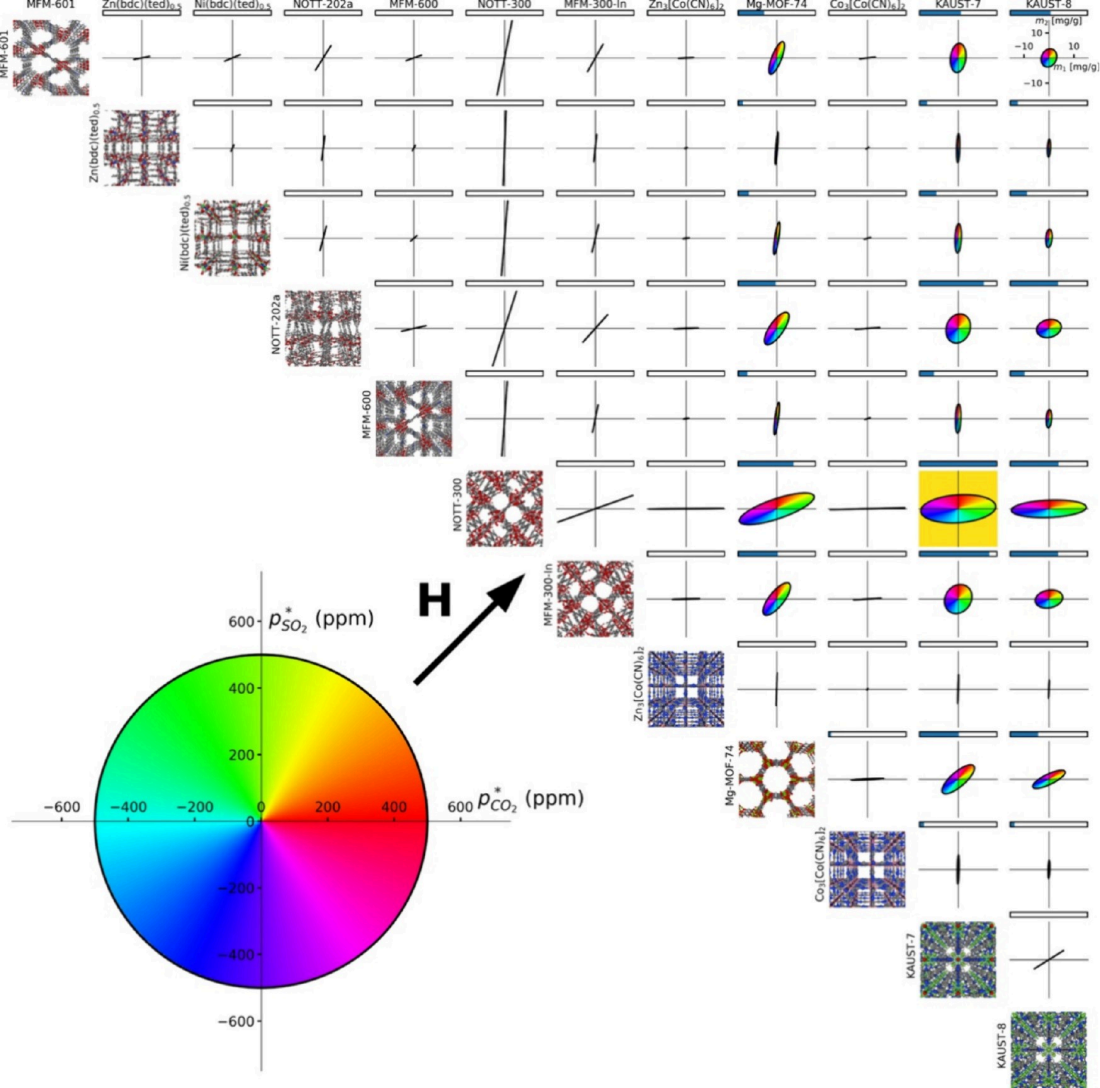
Approach for selecting the optimal MOF combination to create a reliable and accurate gas sensor array given a collection of MOF candidates and their Henry coefficients.^140^ Copyright 2020, American Chemical Society.

There are limited numbers of computational studies on MOF-based sensors in the literature, but the studies are critical in terms of providing theoretical insights into the selection of materials for biomedical gas detection applications. MOFs-based biosensors can be utilized through different approaches such as luminescence, electrochemistry, colorimetry, and surface-enhanced Raman scattering (SERS), which are based on luminescence change, electrochemical property difference, color change, and strengthening of electromagnetic field of MOFs.^136^ The heart of an artificial biological nose is a gas-sensing array, which functions like an electronic nose. Wilmer’s group^137−139^ developed computational methods to assess the sensing performance of electronic noses based on MOF. They defined a sensor array score to measure how well the sensor array composed of different MOFs can differentiate between two gas mixtures based on their response when the gas composition changes and found that the optimal sensor combination depends on the operating conditions. For testing experimentally, they introduced noise into their simulated sensor readings and then used these noisy measurements to predict the composition of the gas mixture, which was already known. Computational design of MOF-based gas sensing arrays for SO_2_ and CO_2_ detection was studied by Sturluson et al.^140^ They created a matrix in which each row represents a material and each column represents its response to a specific gas, which is taken into account with the Henry coefficients of SO_2_ and CO_2_ (the linear map of a circle in their composition in Figure 9). Figure 9 shows a visual high-throughput screening of MOF pairs for a sensor array by creating a matrix for all 66 possible pairs. By applying Singular Value Decomposition (SVD), they identified high singular values (diagonal elements in the matrix) that indicate promising materials with high sensitivity to these gases. Herein, SVD was used to systematically identify the most promising MOF for a sensor array by analyzing and ranking their responses to SO_2_ and CO_2_ (panels in upper diagonal), which can significantly improve the performance of the array. The best MOF pair to form a robust sensor array is {KAUST-7 and NOTT-300}, which are highlighted in yellow in Figure 9 because this combination gives the largest eigenvalue of all pairs.

### 4.4 High-Throughput Computational Screening (HTCS)

The studies we reviewed so far focused on a limited number of MOFs, at most 10. HTCS, which examines a large number of MOFs through molecular simulations, offers a unique opportunity to identify the promising material candidates among many and to develop Quantitative Structure–Property Relationships (QSPR) which link structural characteristics of materials to their functional properties. Results of HTCS studies allow researchers to identify promising materials that exhibit desirable properties such as large surface area, porosity, and chemical stability for drug storage and delivery applications. We summarized the current HTCS studies performed for drug storage, delivery, and separation and focused on at least 20 MOFs in Table 4.

**Table 4:** High-Throughput Computational Studies on MOFs for Drug Adsorption, Drug Diffusion, and Uremic Toxin Separation Applications

Application	# of MOFs studied	Promising candidate(s)	Ref
Ibuprofen, caffeine, urea adsorption	24	IRMOF-74-X	(141)
Amlodipine adsorption	28	IRMOF-74-III	(142)
		IRMOF-3	
		CDMOF-1	
Breath gas adsorption (CO_2_, Ar, NH_3_)	50	ZIF-8	(143)
		XUKYEI	
		MOF-5	
		CMOF-4b	
		MOF-399	
Uremic toxin separation	60	OREZES	(144)
		BEPPIX	
Uremic toxin separation	315	Bio-MOF-11	(145)
		Bio-MOF-12	
		KEXDIB	
Rifampicin, isoniazid, pyrazinamide adsorption	500	BioMOF-100	(146)

Erucar and Keskin^141^ investigated ibuprofen, caffeine, and urea sorption in 24 biocompatible MOFs using GCMC simulations. RAVXIX (IRMOF-74-IX) exhibited the highest uptake of ibuprofen (2.559 g/g), caffeine (3.258 g/g), and urea (2.938 g/g) thanks to its large pore apertures (∼54 Å) and high pore volume (3.7 cm^3^/g). Liu et al.^142^ studied adsorption of an antihypertensive drug amlodipine in 28 MOFs by using GCMC simulations and reported that IRMOF-74-IX exhibited the highest amlodipine uptake (2.315 g/g) at 310 K. Since ammonia is a known biomarker for kidney disease, Wilmer’s group^143^ developed mass-based gas sensor arrays optimized for the detection of kidney disease from breath using GCMC simulations. They computationally screened 50 MOFs from the Computation-Ready, Experimental MOF (CoRE-MOF-2014) database including 5109 MOF structures to find promising MOFs for the detection of kidney disease from breath containing CO_2_, Ar, and NH_3_ gases. GCMC simulations were performed to calculate combined linear adsorption coefficients (CLACs) for each gas/MOF pair, and five promising MOF candidates, ZIF-8, XUKYEI, MOF-5, CMOF-4b, and MOF-399, were reported for the breath sample. Yıldız et al.^145^ performed a computational screening study to remove uremic toxins including creatinine, urea, and water from blood of chronic kidney diseases by MOFs using GCMC simulations.

**Figure 10:**
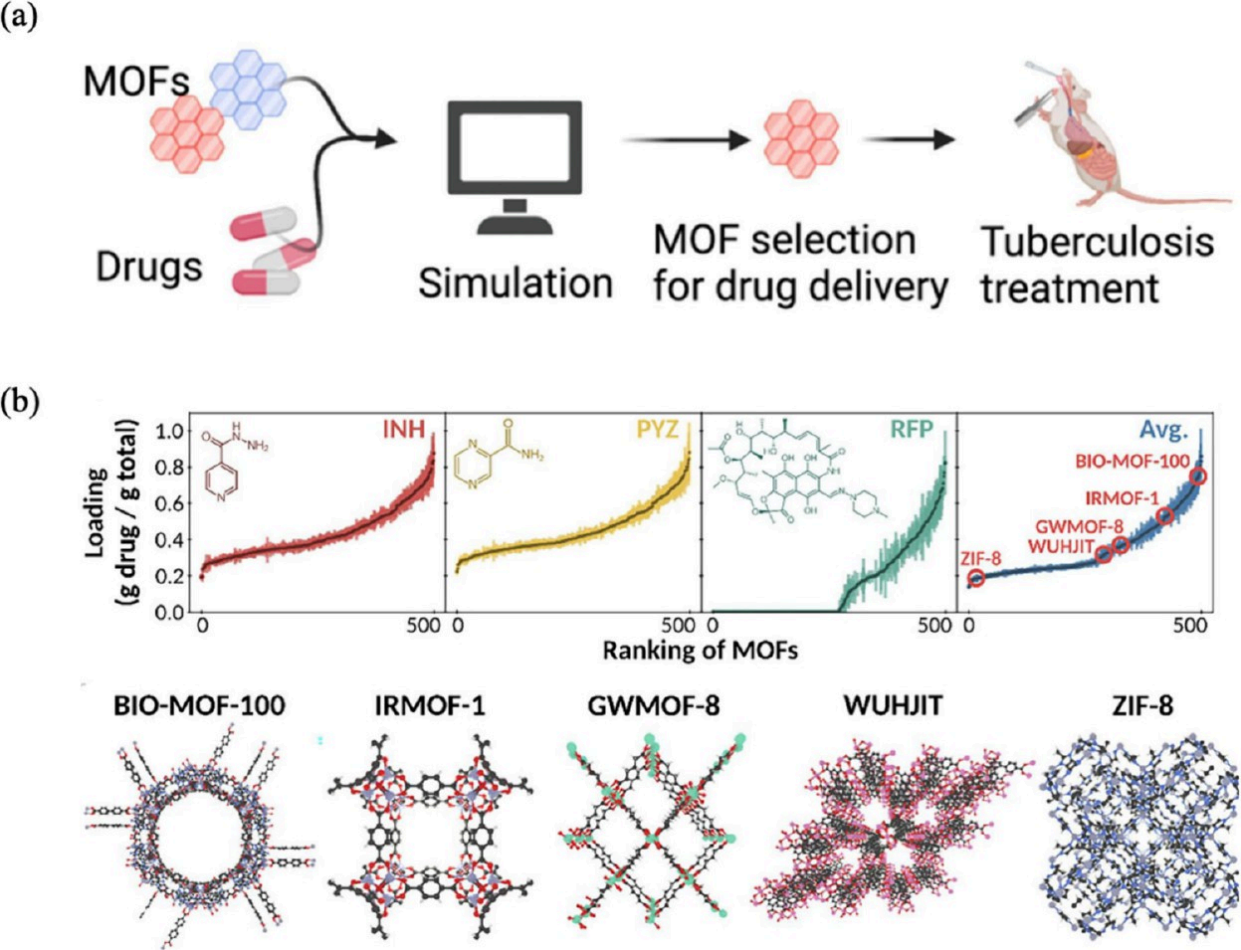
(a) The workflow of selection of MOFs for antituberculosis drugs. (b) Theoretical drug loading capacity for top 500 MOFs with the structures of five selected MOFs namely, BIO-MOF-100 (highest loading), IRMOF-1 (high loading), GWMOF-8 (medium loading), WUHJIT (medium loading), and ZIF-8 (low loading).^146^ Copyright 2022, Elsevier.

A biocompatible MOF data set^147^ composed of 315 MOFs was studied, and Bio-MOF-11, Bio-MOF-12, and KEXDIB were found to be promising for both creatinine/water and urea/water separations. The same group^144^ then investigated membrane-based uremic toxin separations using 60 biocompatible MOFs by GCMC and EMD simulations, and OREZES was found to exhibit the highest membrane selectivity of 347.9 for urea/water separation. Acharya et al.^146^ performed HTCS of 500 MOFs for storage of three antituberculosis drugs (rifampicin: RFP, isoniazid: INH, and pyrazinamide: PYZ) using GCMC simulations. 5109 MOFs from CoRE MOF database were examined and GCMC simulations were performed for the top 500 MOFs with the highest pore volumes among materials with metal centers having relatively lower biotoxicity to calculate the average drug loading. The schematic of the process for selection of five MOFs considering different drug loading capacities for these drugs is given in Figure 10a. Bio-MOF-100 exhibited the highest loading as 63, 72, and 72 wt % for RFP, INH, and PYZ, respectively, as shown in Figure 10b. Bio-MOF-100 was then synthesized and tested in *in vivo* experiments, and results showed the sustained release of RFP, INH, and PYZ. Experimentally determined Bio-MOF-100 loading of 72 ± 8 wt % for INH was in good agreement with its simulated loading, whereas experimental results of 46 ± 9 wt % loading RFP and 53 ± 8 wt % loading PYZ were found to be lower than simulated values.

### 4.5 Machine Learning

Since the number of both synthesized and computer-generated (hypothetical) MOFs is indefinitely high and growing exponentially, molecular simulations require tremendous computational power and resources. The utilization of machine learning (ML) to expedite the MOF selection process has become more prominent, specifically for the HTCS studies on MOFs. The reader is refereed to review articles for the details of using ML in the MOF field.^148−150^ Combining molecular simulations, data science, and experimental validation holds significant promise for advancing the development of new MOFs with tailored properties for biomedical applications, and the field is just starting. There are currently two ML studies on biomedical applications of MOFs: ML models were developed to predict the ibuprofen loading capacity of MOFs.^151^ Data related to metal ions, organic linkers, functional groups, surface area, pore volume, and ibuprofen loading capacities of MOFs were collected from 100 different publications, and ibuprofen loading capacities of 9 MOFs were predicted using organic linkers, metal ions, and functional groups and easily calculable structural properties such as surface area and pore volume as descriptors. Results revealed that the structural properties of MOFs such as pore volume and surface area are the most important features that affect their drug uptakes.

It is important to note that, for biological use of MOFs, the concentrations of organic solvents and metal ions must be kept below safe limits.^152^ Ideally, metal cations with a higher permissible daily intake should be selected to prevent toxicity. Mg, Ca, Fe, and Zn metals stay in the safe zone for drug delivery applications. Considering these facts, Jimenez et al.^153^ investigated the biocompatibility of MOFs utilizing ML and constructed a pipeline aimed to accelerate the evaluation of potential MOF toxicity by using the chemical characteristics of precursors used in the MOF synthesis. They created an extensive bio-MOF database that lists the toxicity of MOF metallic centers by curating a database documenting median lethal dosage (LD50) value of related ions in oxidation states frequently encountered in MOFs. In addition, ML models were developed using LD50 values through a library of more than 35,000 organic compounds, where the best model was 83% accurate in forecasting the possible toxicity of MOF linkers by classifying them as “safe”, “toxic”, and “fatal”. Using these ML models, they were able to screen 86,000 nondisordered MOFs in the CSD to find potential biocompatible MOF candidates for future biomedical applications. In addition to facilitating high-throughput screening, the developed ML models provided insight into the chemical environment surrounding the precursor molecules that are highly biocompatible.

Finally, the metal and linker components of the inorganic building units for the MOFs discussed in this review are summarized in Table 5 below.

**Table 5:**
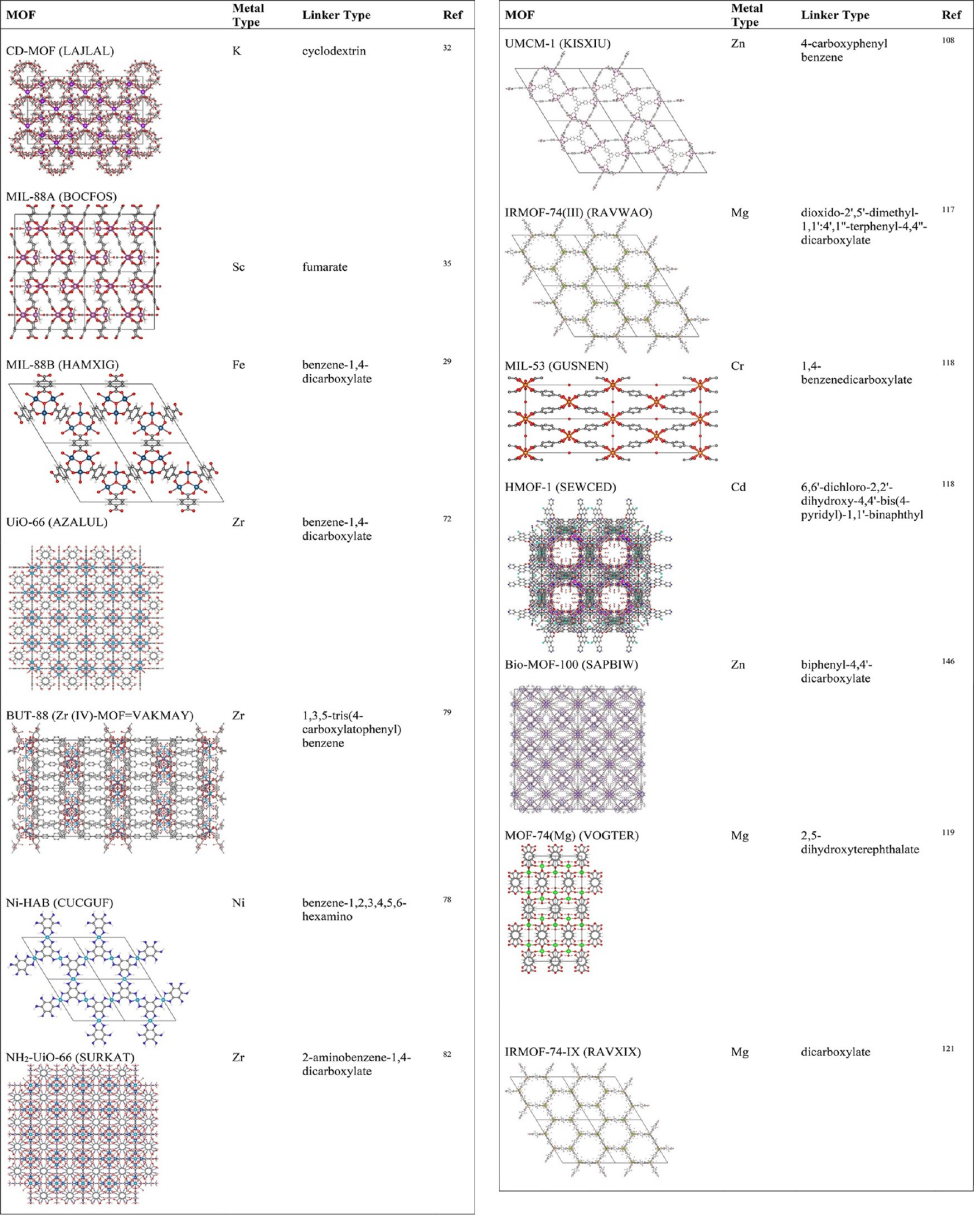

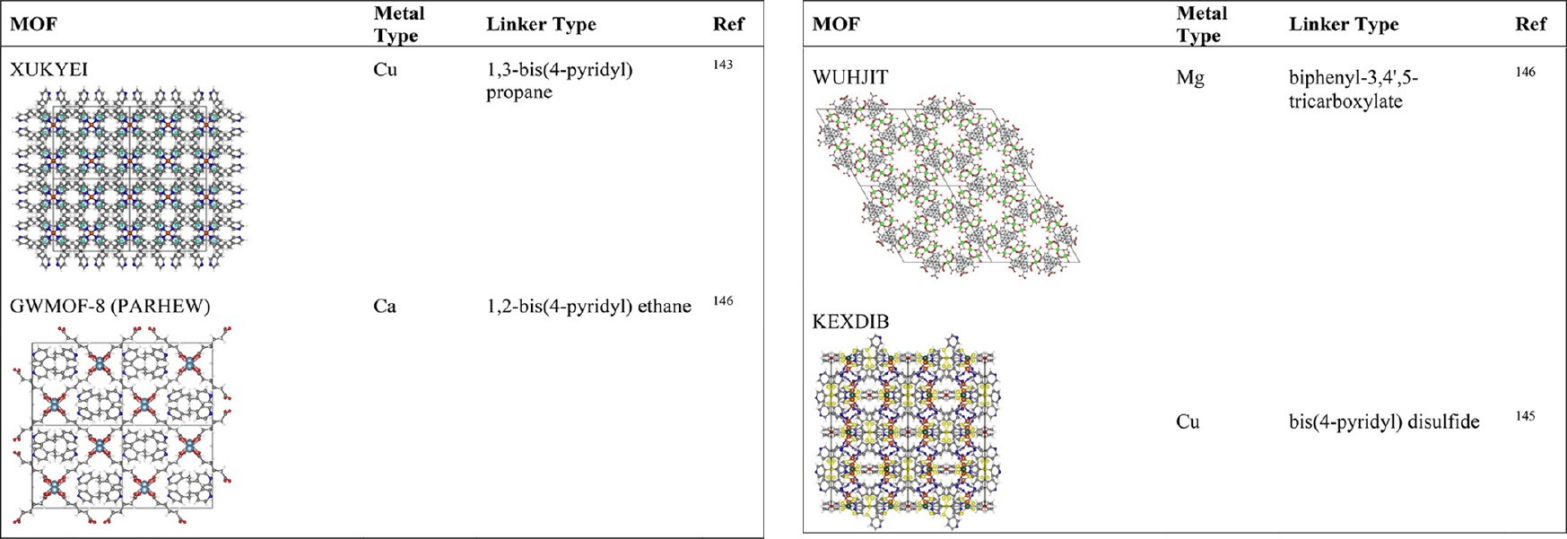
Structure Representation, Metal, and Linker Type of the MOFs Mentioned in This Review^a^

a Gray, white, red, magenta, purple, dark blue, light blue, blue, dark purple, green, orange, pink, brown, and turquoise spheres represent C, H, O, K, Sc, Fe, Zr, Ni, Zn, Mg, Cr, Cd, Cu, and Ca atoms, respectively. Each structure appears as a 2 × 2 × 2 unit cell and is taken from CCDC.^131^

## 5 Outlook

The integration of MOFs into drug delivery systems presents a groundbreaking approach for precision medicine, with the potential to transform therapeutic interventions. Both the experimental and computational studies that we reviewed in this work highlighted that MOFs’ remarkable properties, exceptionally high surface areas, customizable pore sizes, and extensive chemical versatilities collectively enhance their applicability across a broad spectrum of biomedical fields. One of the most significant applications of MOFs lies in drug storage and delivery systems: they can effectively encapsulate the therapeutic agents, which enables a controlled release of the drugs, thereby improving treatment efficacy while simultaneously reducing potential side effects associated with conventional drug delivery methods.^154^

It was shown that MOFs hold significant promise as multifunctional drug delivery platforms, particularly for cancer therapy and Alzheimer’s and Parkinson’s diseases. In cancer treatment, research is progressing toward developing hybrid materials that enable more precise control over drug release, enhance therapeutic efficacy, and reduce systemic toxicity.

For this purpose, the surface of the MOF is typically functionalized with ligands that selectively bind to targets in tumor cells. For example, many studies mentioned folic acid and aptamers as some of the most specific ligands for recognizing cancer cells.^17,45^ Specific targeting could be also achieved by functionalizing the MOF with pH-sensitive molecules such as chitosan, ATP, or glutathione concentration sensitive molecules.^21,36,155^ Additionally, targeted delivery could be facilitated by using pH-sensitive MOFs, such as those from the ZIF series.^42^ Functionalizing MOF surfaces with biocompatible polymers such as polyethylene glycol, chitosan, maltose, or biomolecules (peptides, proteins) can reduce toxicity, improve compatibility with biological systems, and enhance the long-term stability of MOF-based delivery platforms.^17,36^ This functionalization increases resistance to hydrolysis and minimizes leaching of metal ions, which is critical for avoiding toxicity. Combining MOFs with nanoparticles, such as Fe_3_O_4_, ZnO, or GO, can improve their mechanical stability and provide additional benefits, such as enhanced drug delivery or imaging capabilities.^17,37−39^ The biocompatibility of MOFs can also be improved by a promising approach involving the use of biomolecule ligands, such as cyclodextrins, peptides, and amino acids, as well as biocompatible metal ions like calcium, potassium, and sodium, which can mitigate risks associated with MOFs.^46−48^ Several combinations of MOFs and cyclodextrins showed great potential in advancing cancer therapy and personalized medicine. Additionally, the potential for MOFs in multimodal therapy, such as combining chemotherapy with photodynamic or photothermal treatments, opens new possibilities for more effective and minimally invasive cancer therapies.^24^

Regarding neurodegenerative disorders such as AD and PD, MOF-based drug delivery systems show immense promise for advancing treatments of these diseases. The ability to enhance drug solubility, target specific brain regions, and overcome biological barriers, such as the blood–brain barrier, presents significant potential in this field.^32^ Additionally, the combination of MOFs with innovative delivery methods, such as nasal administration and microneedles, provides an effective route to directly access the brain while minimizing systemic side effects. The strategy of repurposing drugs for neurodegenerative conditions represents an efficient path for developing treatments with already established safety profiles.^53^ In PD, MOF-based carriers combined with magnetic nanoparticles open new avenues for targeted dopamine delivery.^35^ These systems, which exploit the nasal pathway to reach the central nervous system, show promise in controlling dopamine release and mitigating the side effects associated with current PD therapies. As research progresses, MOF-based platforms may become essential for noninvasive, precision-targeted treatments that address unmet medical needs in neurodegenerative diseases. Looking ahead, the continued exploration of MOF-based systems for brain drug delivery could revolutionize the treatment of these disorders, improving patient outcomes and the quality of life. Additionally, further exploration of MOFs for other diseases, including antimicrobial therapy and viral diseases, also presents exciting future directions.

Moreover, by functionalizing MOFs with imaging agents, the contrast and clarity of various imaging techniques can be obtained. For biosensing, the unique porous characteristics of MOFs facilitate the detection of biomolecules, even at extremely low concentrations. In tissue engineering, MOFs can serve as scaffolds that not only support cell proliferation but also promote tissue regeneration. The integration of MOFs with biomolecules, such as antibodies, enzymes, and nucleic acids, enhances their performance as biosensors. This combination is vital for the accurate detection of biomarkers, which can significantly improve the prognosis and treatment outcomes. Recent innovations have demonstrated the successful application of MOFs in various biosensing systems such as aptasensors, which utilize aptamers as recognition elements. However, challenges remain, including issues of stability and sensitivity compared with traditional immunosensors. Advances in MOF technology, such as increasing the surface area for aptamer immobilization and utilizing MOFs as redox mediators, have shown promise in overcoming these limitations. For cancer diagnostics, bimetallic MOF-based aptasensors have demonstrated high sensitivity and specificity in detecting cancer cells, while ELISA-MOF-based sensors have enhanced sensitivity and stability in biomarker detection compared to conventional methods. Additionally, the potential of MOFs in catalyzing novel biosensing approaches, such as green electrochemical biosensors for cancer-related miRNA quantification, highlights their versatility and environmental benefits. In the context of AD, MOFs are being tailored to selectively bind AD biomarkers such as amyloid β (Aβ) peptides, leading to the development of fluorescent sensors that offer high sensitivity and wide detection ranges. These advancements illustrate the capability of MOFs to facilitate early diagnosis and monitoring of AD, thereby enhancing patient management. Overall, the unique properties and adaptability of MOFs position them as pivotal components in the ongoing advancement of biomedical technologies, paving the way for innovative treatments and diagnostic tools.

On the other hand, several challenges that we cannot overlooked limit MOFs’ effectiveness in biomedical applications. Various new and efficient synthesis protocols have been recently developed for MOFs, but still these synthesis procedures are mostly complicated and expensive, making it difficult to produce these materials at the large scale for widespread medical use in addition to the complete reproducibility of MOF participles for clinical applications.^156−158^ Studying metal ion interactions with biological ligands for better design and development of simple and cost-effective synthesis methods (considering factors like solvents, temperature, and pH), and improving thermal, chemical, and water stability of MOFs are issues that open to development.^159^ While the high porosity and surface area of MOFs are advantageous for drug loading, they can also result in unpredictable drug release profiles, which may complicate the dosage control, something critical for effective treatment outcomes. One significant concern is the stability of MOFs in biological environments. Many MOFs can degrade when exposed to physiological conditions, which may result in the leaching of toxic metal ions into the body, which may lead to risks to patient safety and adverse effects. Immunogenicity is a further issue as the introduction of foreign materials into the body can trigger an immune response. This reaction can complicate the use of MOFs in therapeutic settings, potentially leading to inflammation or rejection of the material. Without thorough testing and extensive long-term biocompatibility studies, the potential risks associated with MOF use in clinical settings remain largely unknown. Addressing these challenges is essential for the successful integration of MOFs into biomedical applications and can accelerate the transition of MOFs from laboratories to market-ready MOF-based products.

Experimental techniques provide direct evidence of MOFs’ potential and performance in biological systems, ensuring practical relevance. They enable the observation of key properties, such as drug loading, release kinetics, and biocompatibility. Experiments are also crucial for regulatory approval as they assess safety, efficacy, and potential toxicity of the materials. However, these studies are very resource- and time-intensive due to the high cost of materials, equipment, and labor. Scaling up from laboratory findings to clinical or industrial applications is difficult, and ethical concerns, particularly around *in vivo* studies, further complicate the process. In this regard, complementing experimental research with computational efforts, specifically using molecular modeling methods, is increasingly critical in this field. These computational tools enable researchers to predict MOFs’ behavior under various physiological conditions, optimize their design for specific drug delivery applications, and identify new strategies to enhance their performance in addition to expediting the identification of promising MOFs, reducing both time and costs associated with the experimental trials. Computational studies can also provide detailed insights into the complex biological interactions of MOFs with biological systems, ultimately improving their safety and efficacy. Of course, the success of theory-driven approaches depends on the accuracy of the underlying models, which are expected to accurately represent complex biological systems.

We strongly think that biomedical applications of MOFs will significantly benefit from the recent and growing usage of artificial intelligence (AI) methods. For example, AI-guided molecular simulations can help in understanding the interactions between MOFs and biological systems, leading to better safety profiles for new biomedical applications. AI can accelerate the design and optimization of MOFs, allowing for the fast search and discovery of new materials with tailored properties for drug storage, delivery, imaging, and sensing. AI algorithms can analyze vast data sets from biomedical research, identifying patterns and correlations that may not be evident through traditional experimental methods. The integration of AI with MOFs can lead to more effective biosensors, enabling real-time monitoring of biological processes and disease markers. Furthermore, AI can facilitate personalized medicine by predicting patient responses to treatments based on MOF-based therapies. Of course, as in the case of use of AI in other disciplines, all these efforts should carefully consider the accuracy, completeness, transparency, interpretability, and privacy of the data. Overall, the future of MOF-based platforms in biomedicine is highly promising with the potential to significantly impact personalized medicine and improve patient outcomes.
